# Cardioprotective effect of extracellular vesicles derived from ticagrelor-pretreated cardiomyocyte on hyperglycemic cardiomyocytes through alleviation of oxidative and endoplasmic reticulum stress

**DOI:** 10.1038/s41598-022-09627-6

**Published:** 2022-04-05

**Authors:** Ceylan Verda Bitirim, Zeynep Busra Ozer, Dunya Aydos, Kardelen Genc, Seyma Demirsoy, Kamil Can Akcali, Belma Turan

**Affiliations:** 1 Stem Cell Institute, Ankara University, Ankara, Turkey; 2 Department of Neurosurgery, Pennsylvania State University, State College, PA USA; 3 Biophysics Department, Ankara University, Faculty of Medicine, Ankara, Turkey; 4 Biophysics Department, Lokman Hekim University, Faculty of Medicine, Ankara, Turkey

**Keywords:** Biophysics, Cell biology, Molecular biology, Cardiology, Medical research, Molecular medicine

## Abstract

Extracellular vesicles (EVs) play important roles in diabetes mellitus (DM) via connecting the immune cell response to tissue injury, besides stimulation to muscle insulin resistance, while DM is associated with increased risks for major cardiovascular complications. Under DM, chronic hyperglycemia, and subsequent increase in the production of reactive oxygen species (ROS) further lead to cardiac growth remodeling and dysfunction. The purinergic drug ticagrelor is a P_2_Y_12_ receptor antagonist. Although it is widely used in cardioprotection, the underlying molecular mechanism of its inhibitory effect on diabetic cardiomyopathy is poorly elucidated. Here, we aimed to understand how ticagrelor exerts its cardio-regulatory effects. For this purpose, we investigated the anti-oxidative and cardioprotective effect of EVs derived from ticagrelor-pretreated cardiomyocytes under DM conditions. To mimic DM in cardiomyocytes, we used high glucose incubated H9c2-cells (HG). HG cells were treated with EVs, which were derived from either ticagrelor-pretreated or untreated H9c2-cells. Our results demonstrated that ticagrelor-pretreated H9c2-derived EVs significantly decreased the hyperglycemia-induced aberrant ROS production, prevented the development of apoptosis and ER stress, and alleviated oxidative stress associated miRNA-expression profile. Importantly, EVs derived from ticagrelor-pretreated H9c2-cells enhanced endothelial cell migration and tube formation, suggesting a modulation of the EV profile in cardiomyocytes. Our data, for the first time, indicate that ticagrelor can exert an important regulatory effect on diabetic cardiomyopathy through extracellular vesicular modulation behind its receptor-inhibition-related effects.

## Introduction

Diabetes mellitus (DM) is a complex disease caused by a complex interplay between genetic, epigenetic, and environmental factors and characterized by metabolic abnormalities, as well. It is well-known that DM causes a variety of micro-, and macro-vascular complications such as cardiomyopathy, atherosclerosis, and coronary heart disease^1^. In diabetic patients, heart failure develops not only because of the underlying coronary artery disease but also because of the multiple pathophysiological and metabolic abnormalities induced by altered glucose metabolism^2^. Primary and secondary prevention of cardiovascular disease (CVD) involves a multifactorial approach to treat the cluster of risk factors including hyperglycemia, hypercoagulation, and hypertension. The clinical outcomes emphasized that the altered systemic and cardiac glucose metabolism directly contributes to cardiac contractility and function in DM patients thereby triggering ventricular dysfunction^3^. The alteration of cardiac function in diabetes occurs through the activation and/or deactivation of several signaling pathways. Those specific signaling pathways play pivotal roles in the regulation of angiogenesis, production of reactive oxygen species (ROS) which are compromising the myocardial contractility, mitochondrial function/structure, oxidative stress, and cellular death mechanisms such as apoptosis and autophagy^4–7^. Therefore, elucidating the mechanisms of cardiometabolic drug actions on these complex signaling pathways is critical to support novel clinical indications.

Ticagrelor is the most successful purinergic drug, which reversibly targets ADP-mediated G protein-coupled (GPCR) purinergic receptor P_2_Y_12_^5, 6^, and has been widely used in patients with the acute coronary syndrome (ACS) and myocardial infarction (MI). Several clinical studies have reported the higher efficacy and lower risk of ticagrelor application in ischemic events such as cardiovascular and coronary heart diseases compared to clopidogrel that is a P_2_Y_12_ receptor antagonist and irreversibly blocks P_2_Y_12_^1, 7^.

The cardioprotective and therapeutic potential of the extracellular vesicles (EVs), such as microvesicles and EVs, is presenting a promising and alternative treatment in cardiovascular diseases^8^. In the last decade, it was indicated that cardiovascular cells, such as cardiomyocytes, endotheliocytes, fibroblasts, platelets, smooth muscle cells (SMCs)-derived EVs play critical roles in mediating inflammatory and coagulative reactions^9^, neovascularization, and cell migration^10, 11^ in patients with stable coronary artery disease (CAD) and atherosclerosis. Previous studies also implied the important role of cardiac parasympathetic ganglionic neurons and mesangial cell-derived EVs in cardiac repair through demonstration of inhibition of apoptosis in hyperglycemia^12, 13^. However, how the conventional cardiovascular drugs modulate cardiovascular cell-derived EVs and how secreted EVs exert their cardioprotective effect has not been investigated in detail yet. Furthermore, ticagrelor, a P_2_Y_12_ receptor antagonist, beyond receptor inhibition, provides pleiotropic effects under different pathological conditions, including both in vitro and in vivo studies^14, 15^. For instance, Casieri and co-workers showed the important benefit of EVS derived from ticagrelor pre-treated human cardiac progenitor cell (h-CPC) hypoxia-induced apoptosis^16^. In addition, the direct effect of ticagrelor on mitochondrial dysfunction through suppressing ER stress and markedly decreasing autophagosome‑dependent apoptosis in insulin‑resistant H9c2 myocytes, expressed P_2_Y_12_ receptors, was also reported^17^.

Therefore, taken into consideration the cardio-regulatory effects of ticagrelor contributing to the extracellular vesicle modulation, we investigated the anti-oxidative and cardioprotective role of ticagrelor pre-treated cardiomyocytes-derived EVs on hyperglycemic cardiomyocytes. For this aim, we have mimicked the DM model by treating the H9c2 cells with high glucose (25-mM). We hypothesized that ticagrelor treatment modulates the EVs released from H9c2 cells via reducing hyperglycemia associated with enhanced ROS production, ER stress, and autophagy. Investigating the modulatory mechanism of ticagrelor as a widely used cardiovascular drug may improve the beneficial use in terms of administration in antiplatelet treatment strategies.

## Results

### Characterization and the levels of particles H9c2-derived EVs

**Figure 1:**
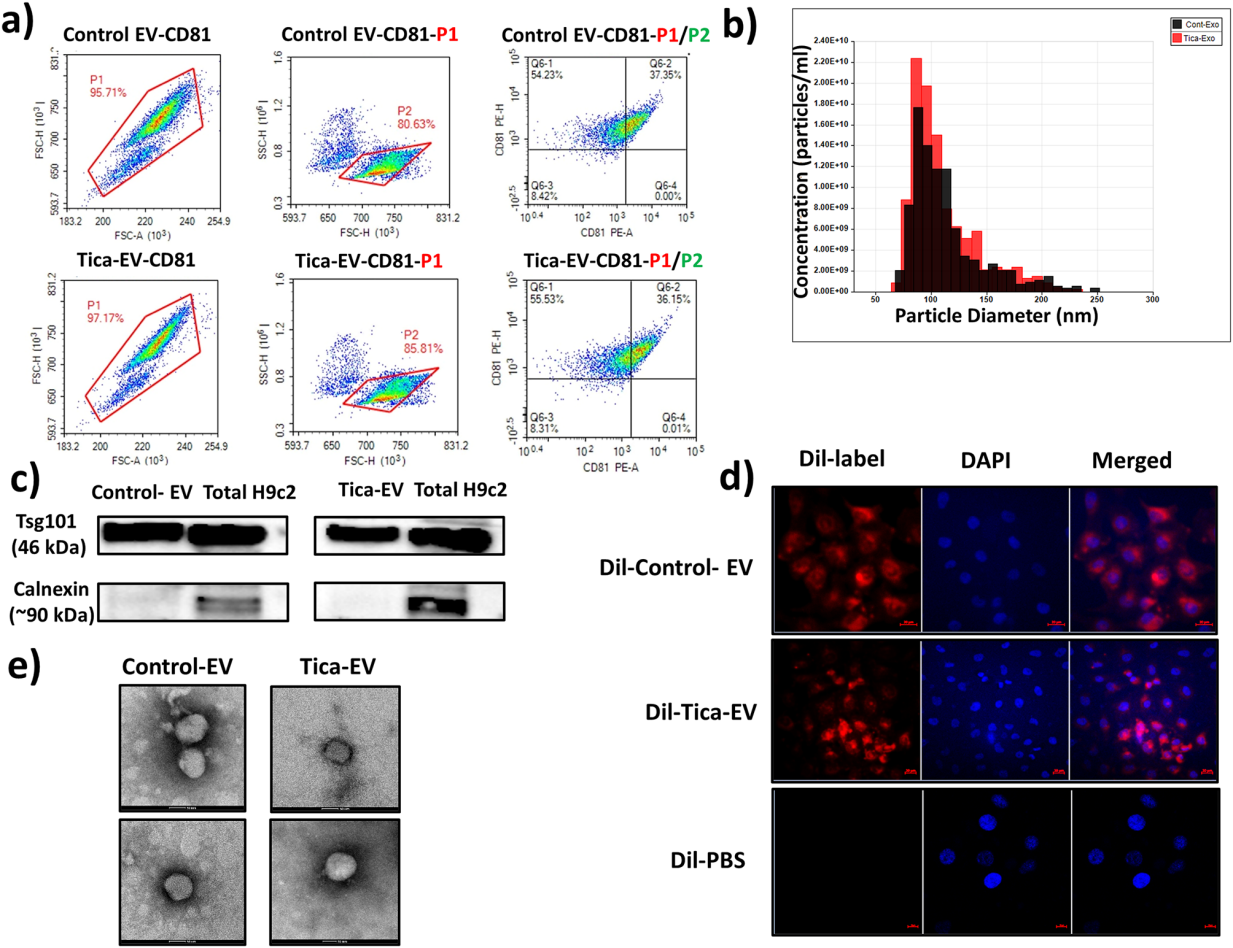
Characterization and quantification of isolated EVs. (**a**) Flow cytometric analysis was performed using magnetic beads coated with CD9 EV marker. These coated beads were incubated with a PE-conjugated CD81 antibody. Analaysis demonstrated as dot plot. FSC represents forward scatter and SSC represents size vs. side scatter. P1 represents gate on the doublet discrimination according to FSC-H vs FSC-A. (**b**) Size distribution and quantification of isolated extracellular vesicles were analyzed using qNanoGold. A polyurethane nanopore rated for particles < 100 Npm (NP100-, Izon Science, UK) was used. The measured mean diameter of EVS was approximately 100 nm. (**c**) Western blot was performed to detect EV proteins and cellular contaminants in isolated EVs. Tsg101 was used as EV markers and endoplasmic reticulum marker Calnexin was used as a negative control. (**d**) EVs isolated from untreated H9c2 (Dil-Control-EV) and ticagrelor treated H9c2 (Dil-Tica-EV) stained with Dil dye. The uptake of DiI-labeled EVs by H9c2 cells was visualized by confocal microscopy. The cells incubated with Dil-labelled PBS are the negative control group. (**e**) EVs isolated from control and ticagrelor treated H9c2 cells were visualized by transmission electron microscopy (TEM).

EVs from both ticagrelor-pretreated and untreated H9c2 cardiomyocytes were characterized with flow cytometry and Tunable Resistive Pulse Sensing (TRPS) technology by qNanoGold. EVs were incubated with anti-human CD9 antibody, EV specific marker-coated latex flow beads. The EV-CD9-bead mixture was detected by another specific marker; PE-conjugated anti-human CD81 antibody. Fluorescence intensity was measured by flow cytometry at 488 nm excitation wavelength (Fig. 1a). Size ranges of EVs were similar in both groups with an approximate size range of 65–240 nm (Fig. 1b). The mean values of control and ticagrelor treated H9c2-derived EVs were measured as 1.82 × 10^10^ particles /ml and 2.25 × 10^10^ particles /ml, respectively.

Isolated EVs are also detected by western blot. EVs were blotted with multivesicular biogenesis marker Tsg101. To detect the contaminant proteins from other cellular compartments such as the endoplasmic reticulum, EV lysate was also blotted with Calnexin. Total H9c2 cellular protein lysate was used as control (Fig. 1c). We also confirmed the EVs uptake of H9c2 cardiomyocytes through incubating the cells with either DiI-labelled or unlabeled EVs (Fig. 1d). Finally, EVs isolated from control and ticagrelor treated H9c2 cells were placed on copper grids, stained with uranyl acetate, and visualized by transmission electron microscopy (Fig. 1e). Note that, their vesicular morphology is similar, and their size range does not exceed 50 nm.

### Ticagrelor-pretreatment modulates the angiogenesis and migration properties of H9c2-derived EVs

**Figure 2:**
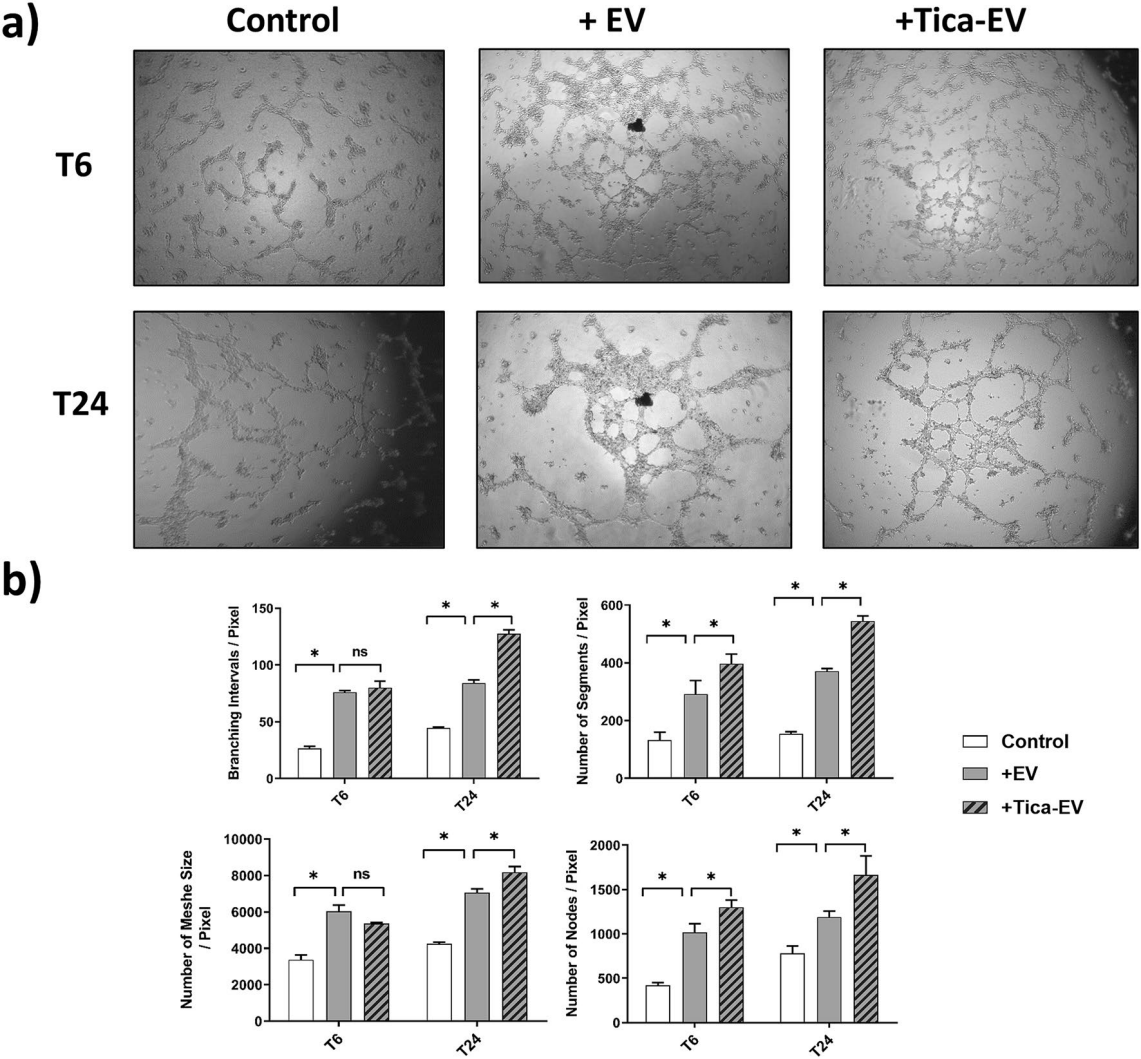
Effects of ticagrelor (Tica, 1 µM) pretreated H9c2-derived EV (+ Tica-EV) on angiogenesis were evaluated by angiogenesis assay using Matrigel. (**a**) The HUVEC cells were seeded on matrigel and incubated either with control (+ EV) or ticagrelor-pretreated H9c2-derived EVS (Tica-EV). Representative images show that Tica-EV treatment causes a marked increase in vasculogenesis. (**b**) The changes in branches, mesh, segment, and node formation of HUVECs at the 6th h and 24th h of incubation were analyzed by ImageJ. Data are expressed as the mean ± SD (n = 3). *p < 0.05, n.s.: not significant.

**Figure 3:**
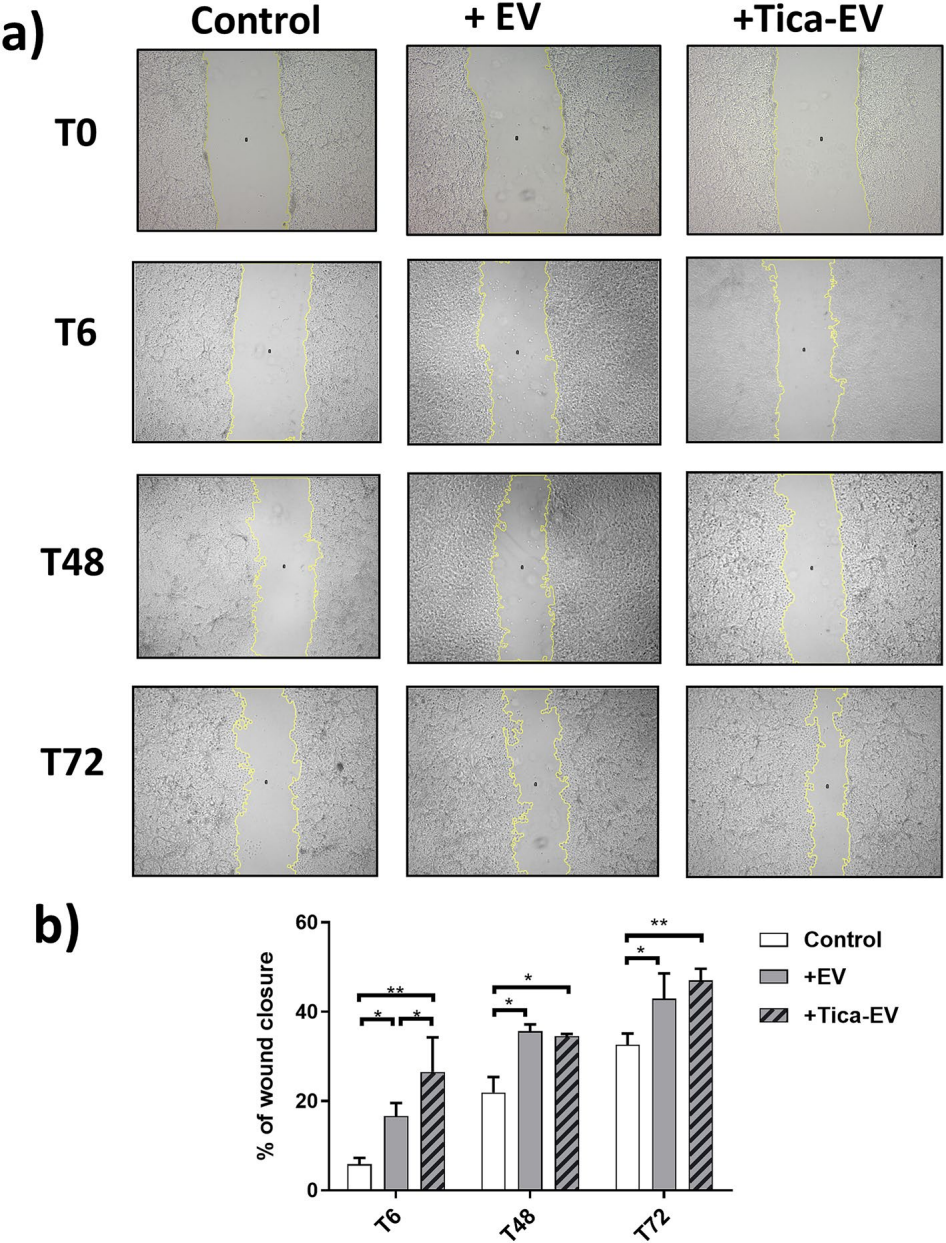
Effects of ticagrelor (Tica, 1-µM) pretreated H9c2-derived EV (+ Tica-EV) on cell migration by wound healing analysis. (**a**) The HUVEC cells were seeded on a 6-well plate and incubated either with control (+ EV) or ticagrelor-pretreated H9c2-derived EVs (Tica-EV). Representative images show that Tica-EV treatment causes a significant increase in wound closure (**b**) The percentage of wound closure is analyzed by ImageJ and 6th h, 48th h, and 72nd h of incubation. Data are expressed as the mean ± SD (n = 3). *p < 0.05, **p < 0.01, n.s.: not significant.

Both to evaluate the functional effects of ticagrelor pretreatment on the modulation of H9c2 derived EVs and the changes in cardioprotective properties of H9c2-derived EV dependent on ticagrelor pretreatment, we have used HUVECs. HUVECs were treated with either ticagrelor-pretreated or untreated H9c2 derived EVs. The changes in the angiogenic and migration ability of HUVEC upon EVs treatment were investigated by tube formation and wound healing assay. As seen in Fig. 2a, ticagrelor-pretreatment induced H9c2-EVs caused a significant increase in branches, mesh, segment, and node formation of HUVECs at the 6th and 24th hours of incubation (Fig. 2b). On the other hand, EV treatment enhanced endothelial cell migration independent of ticagrelor pretreatment (Fig. 3a,b). The significant contribution of ticagrelor pretreated cell-derived EVS in cell migration was observed at T6 (+ EV *vs*. + Tica-EV).

The possible effect of EV treatment on the proliferation of HUVECs, cell proliferation was also tested by MTT assay. There was no significant difference between groups (Supplementary Fig. 1).

### Ticagrelor-pretreated H9c2-derived EVs provide a direct beneficial effect on ROS production in HG cardiomyocytes

**Figure 4:**
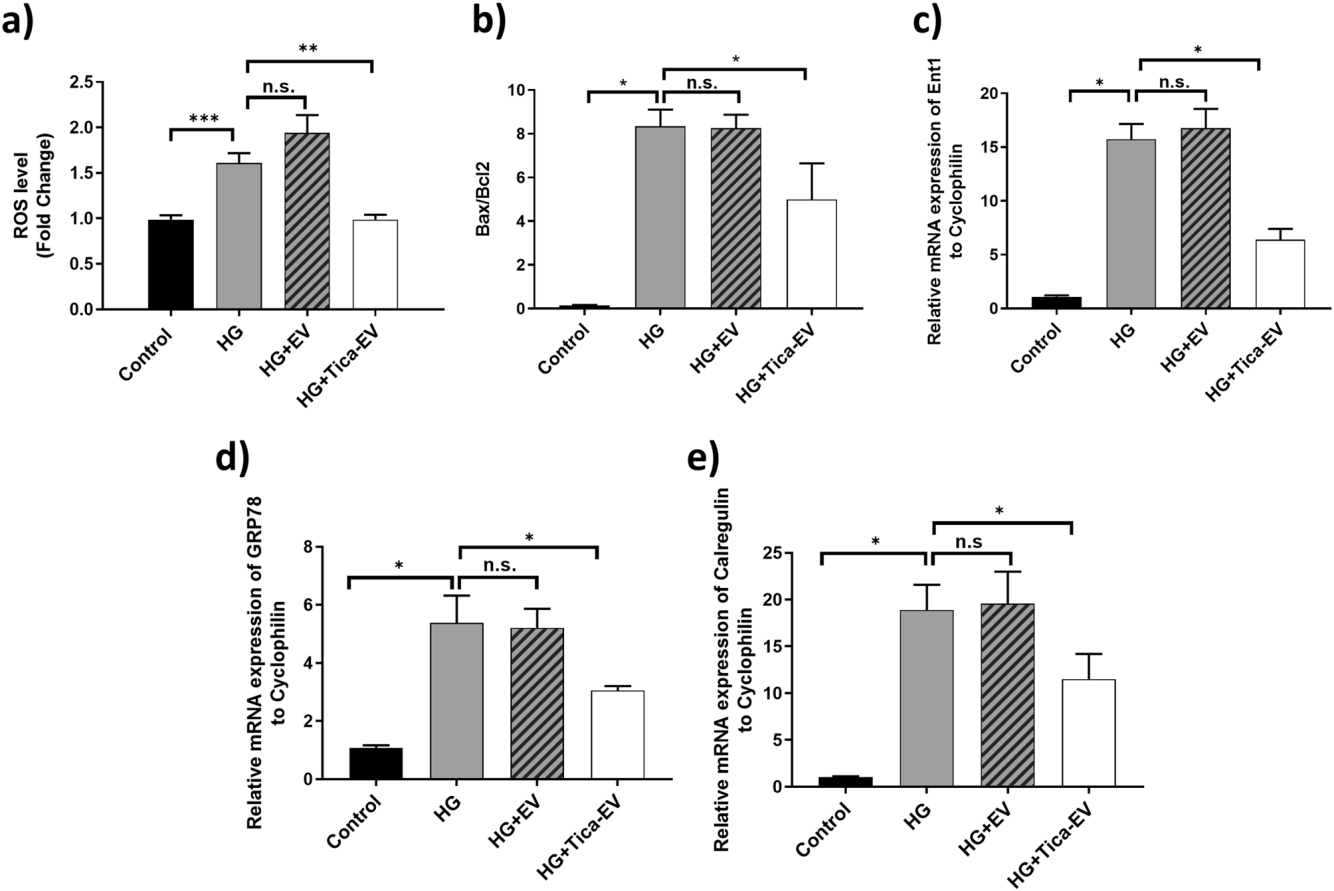
Effects of ticagrelor (Tica, 1-µM) pretreatment of oxidative stress and apoptosis markers. (**a**) The level of ROS production at the cellular level is measured in H9c2 cardiomyocytes. Ticagrelor-pretreated EVs cause a significant decrease in the enhanced ROS production level in HG cardiomyocytes. (**b**) The mRNA expression of Bax and Bcl2 genes are analyzed by qRT-PCR. Bax/ Bcl2 ratio and (**c**) ENT1 is induced upon ticagrelor-pretreated H9c2 EV incubation in HG-incubated H9C2 cells. Ticagrelor-pretreated H9c2 EV treatment reverses the highly increased level of two molecular chaperones: (**d**) GRP78 and (**e**) Calregulin mRNA expressions in HG-H9c2 cells. Data are expressed as the mean ± SD (n = 4). *p < 0.05, **p < 0.01, n.s.: not significant.

In a previous study, it was demonstrated that ticagrelor-treatment markedly prevents the aberrant ROS production in insulin-resistant H9c2 cells^17^. Here, we evaluated the alteration of ROS production of HG cardiomyocytes depending on the ticagrelor-induced H9c2-EV treatment. The response of HG cells to H_2_O_2_ is calculated by a change in fluorescence intensity to represent ROS level at the cellular level. ROS level of HG cardiomyocytes was significantly higher than control cardiomyocytes (p < 0.001). As shown in Fig. 4a, HG cardiomyocytes incubated with control H9c2-EV showed similar ROS levels compared to untreated HG cardiomyocytes, indicating that control EVs do not affect oxidative stress. However, administration of ticagrelor-pretreated EVs caused a significant decrease in the enhanced ROS production level in HG cardiomyocytes.

### Ticagrelor-pretreated H9c2-derived EVs suppress expression levels of apoptosis and ER‑stress markers in HG cardiomyocytes

Treatment of HG cardiomyocytes with EVS from ticagrelor-pretreated H9c2 cells significantly inhibited the ratio of increased Bax/Bcl2 level (Fig. 4b) and equilibrative nucleoside transporter 1 (ENT1) expression (Fig. 4c) compared to untreated HG. In addition to apoptosis markers, EVs from ticagrelor-pretreated HG-H9c2 significantly attenuated the expression levels of ER stress markers. The mRNA level of GRP78 was approximately twofold lower in HG cardiomyocytes treated with ticagrelor-induced H9c2-EVs compared to that of the HG group (Fig. 4d). Similarly, enhanced levels of another ER stress marker, Calregulin was reversed following the administration of ticagrelor-pretreated EVs (Fig. 4e).

### Ticagrelor induced H9c2-EVs inhibits the expression of autophagy markers in HG cardiomyocytes

**Figure 5:**
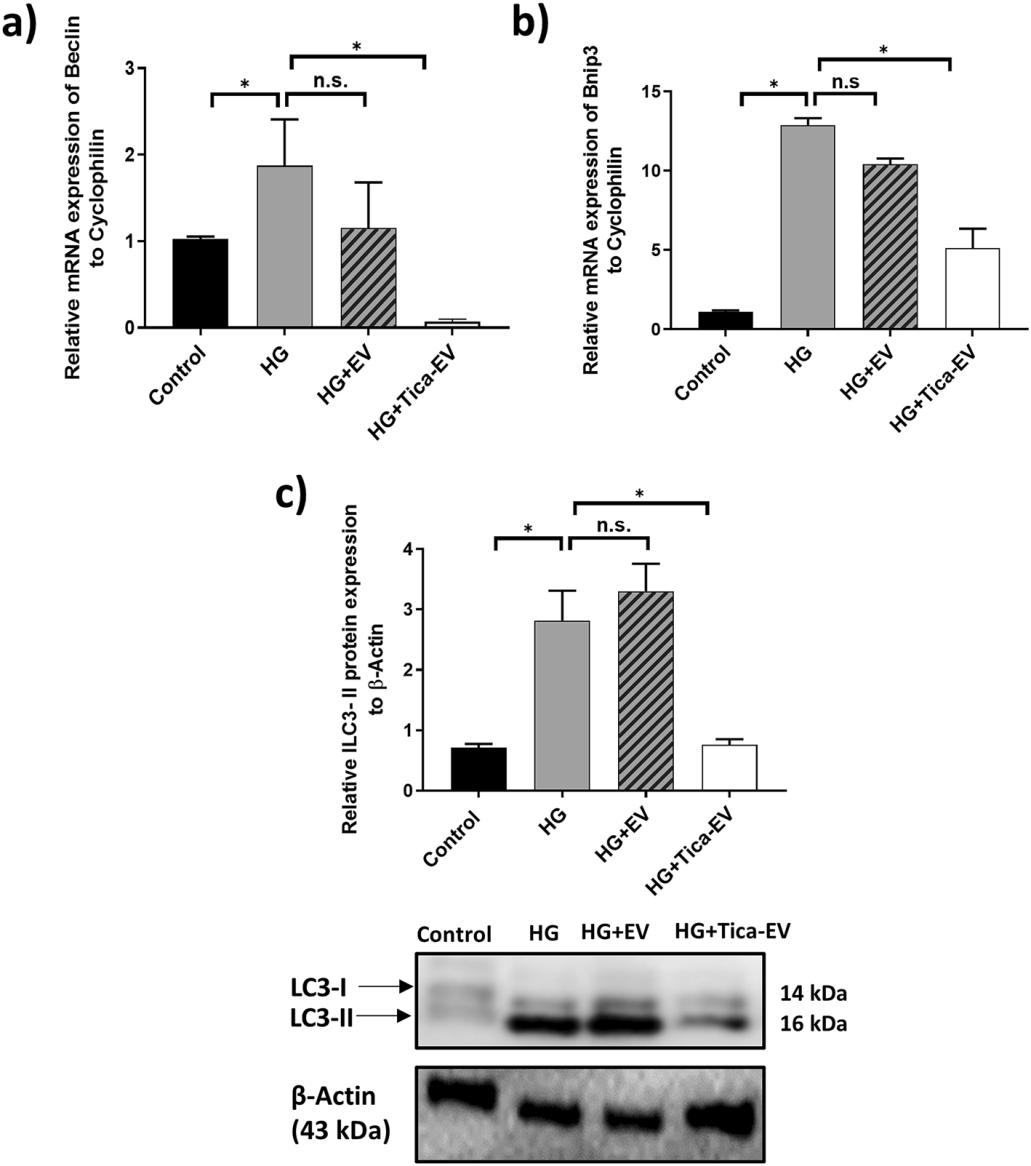
Effects of ticagrelor (Tica, 1 µM) pretreatment on autophagy markers. The changes in mRNA levels of autophagy markers were evaluated by qRT-PCR in control and HG- H9c2 cells. Effects of ticagrelor-treated H9c2 EVs on the mRNA levels of Beclin (**a**), Bnip3 (**b**), and protein level of LC3 (**c**) in HG-H9c2 cells compared to control-EV treated HG-H9c2 cells. LC3 II protein level was normalized to β-Actin and representative Western bands are given in the below of bar-graph. qRT-PCR data are expressed as the mean ± SD (n = 4), Western blot data are expressed as the mean ± SD (n = 3). *p < 0.05, n.s.: not significant.

As shown in Fig. 5, treatment of HG cardiomyocytes with EVs derived from ticagrelor pretreated- H9c2 cells resulted in the inhibition of the expression of autophagy markers Beclin (Fig. 5a) and Bnip3 (Fig. 5b), which are drastically upregulated depending on the hyperglycemia. Microtubule-associated protein light chain 3 (LC3) is widely used to demonstrate autophagy. LC3-II is correlated with the autophagosomes and conversion from LC3-I to LC3-II is detected by immunoblot analysis. LC3-II level of each sample was normalized to β-actin. It is demonstrated that the increased autophagy with hyperglycemia was reversed to almost control level by ticagrelor-induced H9c2-EV incubation (Fig. 5c).

### Ticagrelor induced H9c2-EV upregulates the miR-499, miR-133a, and miR-133b in HG cardiomyocytes

**Figure 6:**
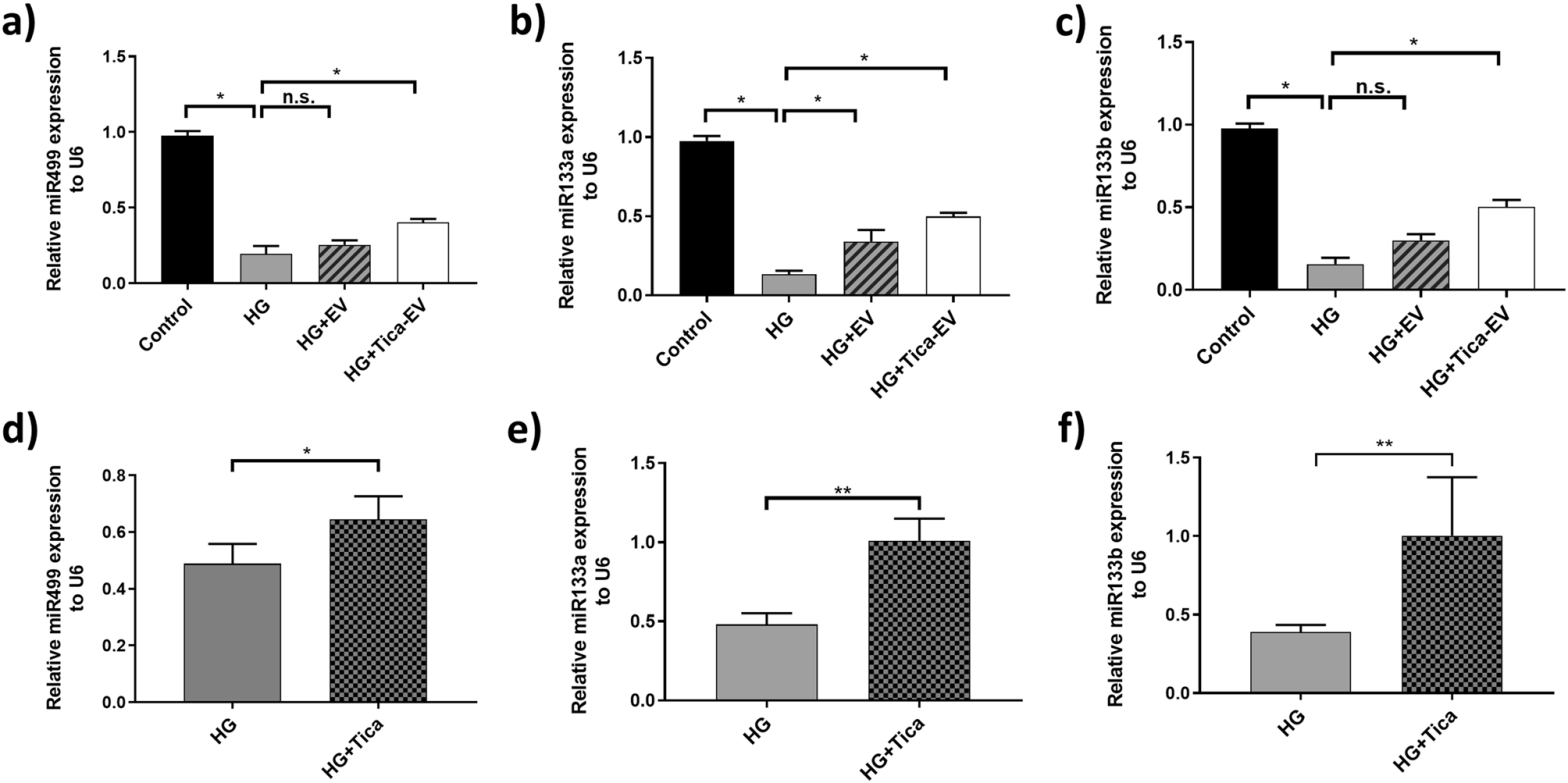
Effects of ticagrelor (Tica, 1 µM) pretreatment on miRNA levels. The mRNA levels of cellular miR-499 (**a**), miR-133a (**b**), miR-133b (**c**) in HG-H9c2 cells compared to control-EV and Tica-EV treated HG-H9c2 cells. mRNA levels of extracellular vesicular miR-499 (**d**), miR-133a (**e**), miR-133b (**f**) HG-H9c2 cells compared to ticagrelor treated HG-H9c2 cells. Data are expressed as the mean ± SD (n = 4). *p < 0.05, **p < 0.01, n.s.: not significant.

We have also evaluated the changes in mRNA level of oxidative stress and cardiomyopathy-related associated miRNAs at the cellular and extracellular vesicular levels. It is well-known that miR-499, miR-133a, and miR-133b are tightly associated with a cardiac action potential, oxidative stress, apoptosis, and cardiomyopathy^19, 20^. Significantly decreased expression levels of miR-499, miR-133a, and miR-133b in DM cardiomyocytes were also reported^21^. First, we demonstrated that downregulated expression of miR-499 (Fig. 6a), miR-133a (Fig. 6b), and miR-133b (Fig. 6c) in DM cardiomyocytes was drastically reversed following the ticagrelor-pretreated H9c2 EV administration.

In addition, to analyze the effect of ticagrelor on DM cardiomyocyte-derived EVs, we have also investigated the changes in the expression level of extracellular vesicular miR-499, miR-133a, and miR-133b. Similar to the expressional changes in cellular miRNA level, extracellular vesicular miR-499, miR-133a, and miR-133b released from DM cardiomyocytes have shown a significant increase in the presence of ticagrelor compared to untreated DM cardiomyocytes (Fig. 6d–f).

**Figure 7:**
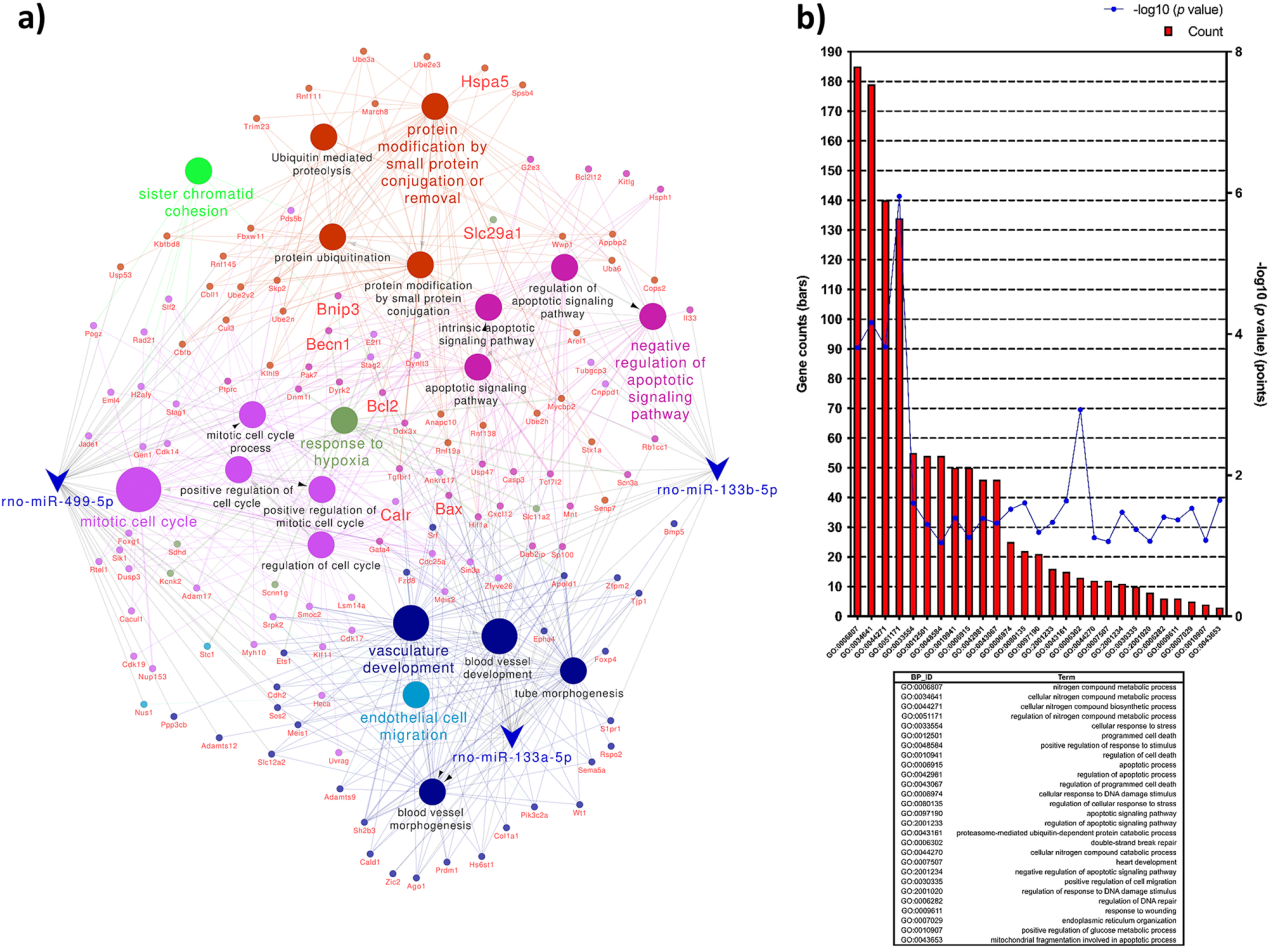
Verification of possible biological processes regulated by the examined miRNAs associated with the pathogenesis of diabetic cardiomyopathy and representative GO-enriched functional annotations for the predicted targets of miRNAs from DAVID. (**a**) The 268 common genes with predicted targets of rno-miR-499-5p, rno-miR-133a-5p, and rno-miR-133b-5p were used to construct miRNA-mRNA regulatory network visualized in Cytoscape. Functional annotation map for the constructed regulatory network was created using ClueGO precompiled source files from GO BP and KEGG databases^18^. (**b**) Database for Annotation, Visualization, and Integrated Discovery (DAVID; v6.8; https://david.ncifcrf.gov) online tool was used to determine the BP attributes of GO for the candidate target genes of miR-499, miR-133a, and mir-133b. GO terms with modified Fisher exact p-value (EASE score) ≤ 0.1, FDR < 0.1, and N per group > 3 were considered strongly enriched.

Next, to verify the biological processes which are associated with the pathogenesis of diabetic cardiomyopathy through miR-499, miR-133a, and miR-133b regulated mRNA dysregulation, GO terms and KEGG pathways enrichment analyses were performed. The 268 common genes were revealed which are the predicted targets of miR-499, miR-133a, and miR-133b. miRNA-mRNA regulatory network of these 268 common genes and four differentially miRNA was used to construct the Cytoscape (Fig. 7a). These 268 common genes and three differentially expressed miRNAs identified in our study were used to construct miRNA-mRNA regulatory network visualized in Cytoscape, which consisted of 386 nodes and 430 edges, including 386 overlapping genes. Among the 412 enriched categories, 20 of them were found to be significantly related to given miRNAs regulation, including mitotic cell cycle, regulation of cell cycle, tube morphogenesis, vasculature development, blood vessel development, blood vessel morphogenesis, apoptotic signaling pathway, response to hypoxia and endothelial cell migration.

GO enrichment analysis results showed that variations in the predicted targets of miR-499, miR-133a, and mir-133b linked with the biological process (BP) were mainly enriched in nitrogen compound metabolic process, cell death, cellular response to stress, regulation of cell death (apoptosis, anoikis), cellular response to DNA damage stimulus, proteasome-mediated ubiquitin-dependent protein catabolic process, heart development, positive regulation of cell migration, response to wounding, endoplasmic reticulum organization, positive regulation of glucose metabolic process, and mitochondrial fragmentation involved in the apoptotic process (Fig. 7b).

## Discussion

In the present study, we demonstrated an important effect of ticagrelor, a drug directly targeting P_2_Y_12_-receptors, on cardiomyocytes-derived EVs in hyperglycemic cardiomyocytes. Our present data emphasized that ticagrelor exerts its regulatory effect on diabetic cardiomyopathy through extracellular vesicle modulation beyond its antiplatelet action.

Among long-term complications of DM, there are both macro and micro-level abnormalities leading to heart diseases besides other complications. DM, at the cellular level, is associated with detectable changes in intracellular function and structure of cardiomyocytes through affecting several molecular mechanisms including oxidative stress, inflammation, apoptosis and autophagy, miRNA regulation, mitochondrial dysfunction, and ER stress^20–22^. Since DM is a major killer worldwide and its very rapid rise emphasizes a serious threat to humans, there are great efforts to develop novel therapeutic approaches for DM management^16, 17, 22^.

DM patients have a high risk for cardiovascular disease, named diabetic cardiomyopathy. Diabetic cardiomyopathy, which has noticeably different pathology from heart failure, was first defined by Rubler et al.^23^, diabetic cardiomyopathy does not include coronary atherosclerosis but is characterized by an irreversible loss of cardiomyocytes, the contractile cellular units of the heart. The study by Wang et al. advances our understanding of the role of cardiomyocyte-EVs and hints at the complexity of extracellular vesicular cargo under diabetic conditions^24^. Here, for the first time in the literature, we have explored the role of ticagrelor-pretreatment of H9c2 myocardium cells, and then on the beneficial effects of EVs derived from them.

Pathophysiological conditions affect several cardiac cell populations, including cardiomyocytes, endothelial cells, fibroblasts, and smooth muscle cells. All these cardiac cell types contribute to pathological remodeling and oxidative stress. Since the glucose uptake process occurs in cardiomyocytes by glucose transporters (GLUTs), changes in cardiomyocyte metabolism may be considered as a leading stimulus for the complex molecular and structural abnormalities such as hypertrophy, mitochondrial dysfunction, oxidative stress, and ER stress. Therefore, we decided to focus on the metabolic and molecular alterations in a rodent cardiomyocyte cell line H9c2 under pathological conditions.

Impaired angiogenesis and endothelial dysfunction are reported as hallmarks of diabetes and coronary artery diseases^25^. It is well known that angiogenesis is a highly controlled mechanism that involves the cell migration and remodeling of adhesion junctions of endothelial cells (ECs) in the heart. Diabetes contributes to endothelial dysfunction, leading to vascular remodeling impaired vasodilation, or increased vasoconstriction^26^. Diabetic patients are known to be at high risk for the development of premature cardiovascular complications^27^. Therefore, understanding the associated mechanism of anti-hyperglycemic and anti-hypoxic drugs in promoting angiogenesis could be of great importance in the management of diabetes-related cardiac diseases. Wound healing (scratch assay) and tube formation (angiogenesis) assays are the most common methods to analyze the changes in migration and vascularization capacities of HUVECs upon EVs treatment. Since the angiogenic contribution of the ticagrelor is known, it is conceivable that ticagrelor may exert this effect via modulating the angiogenic properties of EVS derived from ticagrelor-pretreated cardiomyocytes. We found- for the first time- that ticagrelor-pretreatment markedly induces branches, mesh, segment, and node formation of HUVECs at the 6th and 24th hours of incubation. Although H9c2-derived EVs enhanced endothelial cell migration independently of ticagrelor pretreatment, the significant contribution of ticagrelor in cell migration was only observed at T6 (+ EV vs + Tica-EV). According to these results, we suggest that EV treatment may show its regulatory effect on migration in an early and short time while it promotes tube formation in a longer period.

It is well established that ticagrelor therapy administered in diabetic patients demonstrated a direct beneficial effect against heart dysfunction beyond its antiplatelet action^28, 29^. Olgar et al. reported that ticagrelor provided a significant improvement in mitochondrial membrane potential, a marked decrease in cellular ROS production, a normalization of the increased resting level of cytosolic Ca^2+^, and preservation of cellular ATP production. Moreover, it was also demonstrated that ticagrelor alleviates enhanced ER stress, autophagosomes formation, and apoptosis by inhibiting the expression of the genes involved in this molecular pathways^17^. However, the exact subcellular mechanisms by which ticagrelor exerts its cardioprotective, anti-hypoxic, and anti-apoptotic effect remains to be elucidated. Consistently, Casieri and co-workers have shown that long-term treatment with the lower dose of ticagrelor enhances anti-hypoxic and anti-apoptotic effects of EVS released from human cardiac-derived mesenchymal progenitor cells (hCPC) via attenuating the increase of HIF1α levels in hypoxic cardiomyocytes^16^. This finding revealed a new role for ticagrelor in the regulation of anti-apoptotic and anti-hypoxic properties of EVS derived from hCPCs, which are stem/progenitor cells resident in the adult heart^30^. Taking into consideration, the well-established actions of ticagrelor, particularly, associated with extracellular vesicle modulation in terms of cardiac regeneration and cardiomyocyte death, in this study, we have demonstrated that ticagrelor induced H9c2 EVs have a direct beneficial effect on hyperglycemic ventricular cardiomyocyte causing a marked decrease in cellular ROS production.

Emerging preclinical and clinical evidence demonstrates that diabetes induces cardiomyocyte apoptosis. EVs derived from ticagrelor-pretreated H9c2 cells demonstrated an anti-apoptotic effect on hyperglycemia-induced cardiomyocytes through suppressing the expression of apoptotic genes, Bax and ENT1.

On the other hand, cardiac autophagy is still a controversial issue. In addition to the beneficial effect of autophagy during ischemic reperfusion^31^, uncontrolled excessive induction of autophagy may contribute to autophagic cardiomyocyte death^32^. Bcl-2 family members Bnip3, Beclin, and microtubule-associated protein 1A/1B-light chain 3 (LC3) are key regulators of necrosis, autophagy, and/or apoptosis^33^. Consistent with the previous study^17^, our data demonstrated that the treatment of HG cardiomyocytes with EVs derived from ticagrelor included H9c2 cells causes the inhibition of the expression of autophagy and apoptosis markers, which are drastically upregulated depending on the hyperglycemia. Of note, the control-EV treatment has shown a remarkable effect in induced autophagy formation and apoptosis in HG cardiomyocytes.

Furthermore, it is also well accepted that autophagy was induced as an adaptive response against ER stress since it was sensitive to ER stress inhibition^34^. Supporting these statements, it was reported that ticagrelor can directly affect cardiomyocytes and provide marked protection against ER stress and dramatic induction of autophagosomes, and therefore, can alleviate the ER stress-induced increases in oxidative stress and cell apoptosis during insulin resistance^17, 35^. In the present study, we have shown that ticagrelor exerts this suppressive effect on markedly increased ER stress via modulating released EVs from H9c2 cells which cause the significant downregulation of mRNA levels of ER stress markers. Finally, we have also evaluated the changes in mRNA levels of oxidative stress and cardiomyopathy-related associated cellular and extracellular vesicular miRNAs. miR-133 and miR-499 are involved in the transcriptional and posttranslational regulation of cardiac hypertrophy^36^. Previous studies have also shown that miR-499 and miR-133 are remarkably decreased in isolated ventricular cardiomyocytes from type 1 diabetes-induced rat hearts. Normalization of cardiac function and oxidant/antioxidant level has been restored after N-acetylcysteine (well-known antioxidant) treatment of diabetic rats together with a significant increase in the expression levels of these miRNAs^21^. In addition, in the insulin-resistant modeled cardiomyocytes, it has been also shown an antioxidant-like effect of ticagrelor particularly on high ROS production and depressed ATP production^17^. From this regard, here, we aimed to examine the action behind its receptor-associated effects and therefore bring new insights on future research and clinical applications of this drug. With this aim, we examined the status of these miRNAs in ticagrelor treated hyperglycemic cells to provide an interpretation of our findings on how associated with ROS production and oxidative stress as well as an antioxidant-like action of ticagrelor in these cells. A supporting study has been previously performed and demonstrated that lipopolysaccharide-treated rat cardiomyocytes showed a marked decrease in the expression of miR-499 which inhibited the expression of pro-apoptotic genes and upregulated expression of the anti-apoptotic gene *BCL-X*_*L*_^37, 38^. miR-499, also attenuates doxycycline-induced mitochondrial fission, apoptosis, and cardiotoxicity via the targeting p21^39^. NFATc4 (nuclear factor of activated T cells), a hypertrophy-associated mediator, is a negatively regulated target of miR-133a^40^. It was also observed that mir133a is significantly down-regulated in hypoxic H9c2 cells. Over-expression of miR-133a suppresses hypoxia-induced apoptosis and increases cardiomyocyte survival rate^41^. miR-133b expressed specifically in the skeletal muscle was reported to be downregulated in STZ-induced diabetic rats concerning increased oxidative stress^21^ and protected H9c2 against hypoxia injury via downregulation of nucleotide-binding oligomerization domain-like receptor protein 3 (NLRP3)^42^. Therefore, one can hypothesize that the restoration in the reduced levels of these miRNAs may lead to cardiac protection against diabetes-caused injury^21^.

Considering the important fact that the diabetic heart composes both healthy and diabetic cardiomyocytes, we have also examined the therapeutic paracrine effect of HG cardiomyocytes following the ticagrelor treatment. Extracellular vesicular miR-499, miR-133a, and miR-133b in hyperglycemic cardiomyocytes were upregulated upon ticagrelor treatment. These results supported that ticagrelor treatment may promote the therapeutic effect of EVs released from both healthy and hyperglycemic cardiomyocytes.

In the present study, we have also examined predicted targets of miR-499, miR-133a, and miR-133b linked with biological processes. These miRNAs have been involved as negative regulators of apoptosis, positive regulators of cell migration, wound healing, and cellular stress response. Such biological relevance of target genes elucidated through GO-enrichment may account for the pathogenesis in diabetic cardiomyopathy through their involvement, especially in heart development (GO:0007507) and positive regulation of glucose metabolic process (GO:0010907) (*p* < 0.05). Taken together, this analysis supports the effect of ticagrelor pretreatment on suppression of cellular stress, ROS production, cell death in addition to the contribution of angiogenesis and cell migration via modulation of extracellular vesicle profile.

## Conclusions

Considering widely usage of ticagrelor for clinical therapy, these observations will get a special interest for clinicians to treat patients suffering from cardiac diseases associated with these cellular abnormalities. Our data reveal an unexpected regulatory mechanism of P_2_Y_12_- receptor antagonist, ticagrelor. Here we indicated the regulatory role of EVs secreted by ventricular cardiomyocytes, possibly depending on induction of ticagrelor on the anti-hypoxic and anti-apoptotic signaling pathways in diabetic cardiomyopathy. We assume that elucidating the mechanisms of cardiometabolic drug actions on the complex signaling pathways may provide a promising alternative pharmacological usage to protect the heart against any pathological stimuli.

## Limitation of the study

The major limitation of the study is that no in vivo data were presented to show the therapeutic effect of ticagrelor via regulating paracrine signaling of cardiomyocytes in the diabetic rat. In our study, we did not investigate the changes in extracellular vesicular miRNA profile depending on the ticagrelor treatment. The alteration of these miRNAs leads to cardiac protection against diabetes-caused injury. The miRNA content, in terms of the cardioprotection, may support the present results and may reveal the potential targets of ticagrelor in EV-based cardiomyocyte protection. However, further experiments focusing on the miRNA repertoire should be performed to confirm ours in vitro results supporting wide evidence of ticagrelor-mediated cardioprotection.

## Materials and methods

### Cell culture

The H9c2 cell line was derived from the left ventricle of the embryonic rat heart (CRL1446) and Primary Human Umbilical Vein Endothelial Cells (HUVEC) derived from the vein of the umbilical cord (CRL1730) were purchased from The American Type Culture Collection. H9c2 cells and HUVEC were grown in modified Dulbecco’s modified Eagle’s medium (DMEM) (Biowest, France) including 5.5 mM glucose (low glucose) and DMEM/F12 medium (Lonza, Swiss), respectively. HUVEC media includes 10% fetal calf serum (FBS) (F2442, Sigma-Aldrich, USA), 50 U/ml penicillin (Lonza, Swiss), 2 mM l-glutamine (Lonza, Swiss), 0.1 mg/ml Heparin and 50 μg/ml streptomycin (Lonza, Swiss). H9c2 DMEM low glucose media includes 10% FBS (Sigma-Aldrich, USA), 50 U/ml penicillin (Lonza, Swiss), 2 mM l-glutamine (Lonza, Swiss), and 50 μg/ml streptomycin (Lonza, Swiss).

To obtain hyperglycemic cells (HG-group), H9c2 cells were incubated with DMEM, including 25 mM glucose (Biowest, France). 1-μM of ticagrelor was applied for 72 h to ticagrelor treated groups^17, 43^. Both ticagrelor treated and untreated groups were incubated in DMEM media including 1% EV-free FBS, 50 U/ml penicillin, 2 mM l-glutamine, and 50 μg/ml streptomycin for the EV isolation.

**Figure 8:**
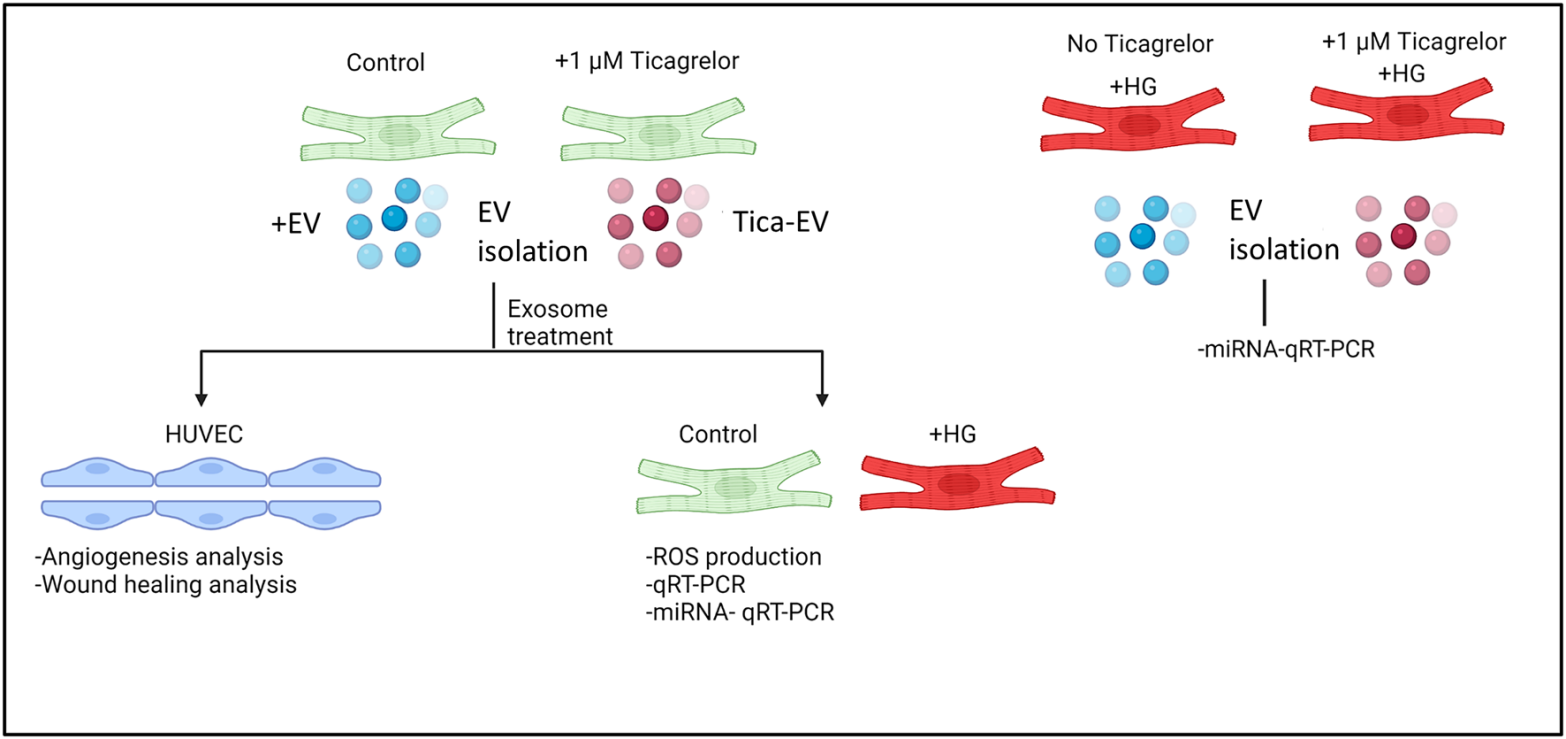
The followed experimental model and protocols in the present study. The Diabetes model was mimicked using the H9c2 cell line. EVs derived from ticagrelor treated and untreated H9c2 cells were analyzed at the functional and molecular levels. The image was drawn using BioRender (https://biorender.com/).

Control and HG cells were treated with 0.5 ug EV isolated from either control (+ EV) or ticagrelor treated H9c2 cells (Tica-EV) for 48 h. During the EV treatment, the cells were incubated in DMEM media including 1% EV-free FBS, 50 U/ml penicillin, 2 mM l-glutamine, and 50 μg/ml streptomycin. The experimental protocol followed step by step was given in Fig. 8.

### Extracellular vesicle isolation

EVs were isolated from the supernatant using qEVs T Size exclusion chromatography (SEC) columns (Izon Science, Poland) according to the manufacturer’s protocol. Briefly, the collected supernatant either from ticagrelor-treated or control H9c2 cells was centrifuged at 10,000xg for 10 min, and the supernatant was added into columns. The particles were separated and purified optimum between at 35 and 350 nm range. The collected particle suspension was centrifuged at 100,000×*g* for 1 h to increase the concentration.

### Extracellular vesicle characterization and quantification

EV characterization was performed by flow cytometry and western blotting analysis. Firstly, latex beads (Thermo Fisher, USA, Cat no: 37278) were coated with EV- specific anti-human CD9 antibody (Biolegend, SanDiego, Cat no: 312102) overnight at 4 °C to capture EVs that have been isolated from size exclusion chromatography (SEC) columns. For this purpose, 1 µg EV were incubated with CD9-coated beads overnight at 4 °C. Bead-antibody-EV mixture was washed with 1xPBS to remove the unbound EVs and then stained with PE-conjugated anti-human CD81 antibody (Biolegend, SanDiego, Cat no: 104905). CD-81-stained EVS were detected by flow cytometry. The doublet discrimination was done according to FSC-H vs FSC-A dot plot. After the exosome-bound bead population was selected, the percentage and median fluorescence intensity (MFI) of positive cells were determined considering the autofluorescence control.

For immunoblotting, the concentration of EVs was evaluated by Micro BCA assay (Thermo Fisher, USA). The protein lysate of EVs was loaded to 12% SDS-PAGE gel. Following the transfer and blocking steps, the membrane was incubated with Tsg101 (Abcam, Cat no: ab125011), Calnexin (Abcam, Cat no: ab133615). Anti-rabbit (Abcam, Cat no: ab150077) was used as a seconder antibody.

The size distribution and quantification of isolated EVs were also measured using qNanoGold. For EV samples, a polyurethane nanopore rated for particles < 100 nm (NP100-, Izon Science, UK) was used.

Internalization of EVs into H9c2 cells was also validated by confocal microscopy. 10 µg/ml EVs were dissolved in 1× PBS to a final volume of 200 µl. EVs were stained with Dil (Thermo Fischer, USA, Cat no: D3911) dye. 1× PBS was also labeled with the same concentration of Dil-dye to use as a negative control. Incubation was performed by rotation for 1 h at room temperature in the dark. Following the labeling procedure, the labeled EV and PBS suspensions were filtered using 50 kDa MWCO filters (Merck) to remove the unbound dye. After the centrifugation at 3000 rpm for 30 min, EVs were collected on the filters with 1× PBS containing 1% BSA. H9c2 cells were seeded on a 12-well plate at a concentration of 3 × 10^5^/ml were treated with 5 µl of either labeled-EV or labeled-PBS and incubated at 37 °C. The coverslips were mounted with mounting media with DAPI. Visualization was performed by confocal microscopy at 549 nm excitation wavelength (Zeiss, Germany). EVs derived from control and ticagrelor treated H9c2 cells were placed on copper grids and negatively stained with 2% uracyl acetate after removing the extra moisture. Isolated EVs were visualized under a transmission electron microscope (TEM). Cup-shaped structures, with 30–100 nm sizes were identified as being EVs. The TEM image was acquired at Middle East Technical University (METU) Central Lab.

### Wound healing assay

Endothelial cell migration was evaluated by the scratch assay, as previously reported^44^. Briefly, a total of 1 × 10^5^ HUVECs were seeded in DMEM-F12 starvation medium with 0.5% FBS for 24 h in 6-well plates and allowed to form a monolayer overnight at 37 °C in a 5% CO_2_ incubator. Using a p200 pipette tip, scratches were made in each well of the confluent monolayer. The medium was changed with DMEM-F12 including 1% EV-free FBS and the cells were treated with either 0.5 µg EVs derived from control or ticagrelor treated H9c2 cells. The wound closure was visualized at 6 h, 48 h, and 72 h of incubation by an inverted light microscope (Zeiss, Germany). Migration was quantified by measuring the width of the cell-free zone by wound healing Analyzer plug-in for ImageJ (NIH, USA)^45^. The changes in the migration of EV-treated cells were expressed compared to (untreated) controls as the pixel. The equation is the following:

$$\% {\text{ of wound closure}} = {{\left( {\left( {{\text{A}}^{{{\text{TO}}}} {-}{\text{A}}^{{{\text{TX}}}} } \right) \times {1}00} \right)} \mathord{\left/ {\vphantom {{\left( {\left( {{\text{A}}^{{{\text{TO}}}} {-}{\text{A}}^{{{\text{TX}}}} } \right) \times {1}00} \right)} {{\text{A}}^{{{\text{TO}}}} }}} \right. \kern-\nulldelimiterspace} {{\text{A}}^{{{\text{TO}}}} }}$$

here, A is the area, T0 is the time zero (at the time EV were added), TX is the time at distance was calculated (T6, T48, T72).

Values represent the mean (± SD) of triplicate scratches.

### Tube formation assay

Tube formation assay was performed as previously reported^46^. Briefly, a total of 35 × 10^4^ HUVECs were seeded in Matrigel-coated (Corning, USA) 96-well plates in DMEM-F12 including 1% EV-free FBS. The cells were treated with either 0.5 µg EVs derived from control or ticagrelor treated H9c2 cells. The tube formation was visualized at 6 h and 24 h of incubation by an inverted light microscope (Zeiss, Germany). Branche, mesh, segment, and node formations were analyzed by Angiogenesis Analyzer plug-in for ImageJ (NIH, USA)^47^. Changes in angiogenesis on EV-treated cells were expressed as a percentage of the controls (untreated cells). Values represent the mean (± SD) of triplicate formation assay.

### ROS measurements by confocal microscopy

Cells were loaded with 10 μM ROS indicator chloromethyl-2′,7′-dichlorodihydrofluoroscein diacetate (DCDFA) probe (Sigma-Aldrich, USA Cat no: D6883) by incubating cells for 60 min at room temperature. Probe-loaded cells were scanned by a confocal microscope (Leica, TCS SP5, Germany) at ~ 490–520 nm. To obtain maximal fluorescence intensity associated with ROS production, the cells were exposed to H2O2 (100-μM), acutely. The fluorescence intensity changes for every cell were calculated by Δ*F*/*F*0, where Δ*F* = *F* − *F*0; F is identified as local maximum elevation of fluorescence intensity over basal lEVsel, *F*0.

### The measurement of mRNA levels of cellular and extracellular vesicular miRNAs by qRT-PCR

To analyze mRNA levels of cellular miRNAs, total RNA of H9c2 cardiomyocytes was isolated by using RiboEx Reagent (GeneAll, Korea) and the purified total-RNA was reverse transcribed with Entlink cDNA Synthesis-kit (Elk Biotech., Wuhan), as described previously^48^. MicroRNA isolation from EVs was performed using the total extracellular vesicular RNA isolation kit (Thermo Fischer; Cat no: 4478545). For miRNA qRT-PCR, stem-loop RT primers, that were designed for each miRNA, were used for cDNA Synthesis^49^. Shortly, GoTaq^®^ qPCR Master Mix (Promega, A6001) was used to quantify and amplified PCR products for each primer, and primers’ specificity was controlled with known databases. The fold changes of genes were analyzed based on the comparative (2^− ΔΔCt^) method. Cyclophilin was used as housekeeping control for mRNA expression analysis and U6 was used as housekeeping control for miRNA expression analysis. For miRNA-qPCR, the universal reverse primer sequence; 5′-CCA GTG CAG GGT CCG AGG TA-3′ was used as antisense^50^. The miRNA and mRNA primers designed for qRT-PCR are given in Supplementary Table 1, and stem-loop RT primers designed for each miRNA are given in Supplementary Table 2.

### Western Blot for LC3 detection

Total proteins from control H9c2, HG-H9c2, EV-treated HG- H9c2, and Tica EV-treated HG-H9c2 cells were isolated. Protein lysates were evaluated by BCA assay (Thermo Fisher, USA). 30 µg protein from each group was loaded to 15% SDS-PAGE gel. Following the transfer and blocking steps with 1% BSA in TBS-0.3% Tween, the membrane was incubated with LC3 (Abcam, Cat no: ab63817) primer antibody and β-Actin (Santa Cruz, Cat no: ab1615) primer antibody which was used as house-keeping control. Anti-rabbit (Abcam, Cat no: ab150077) and anti-goat (Santa Cruz, Cat no: sc-2020) antibodies were used as seconder antibodies, respectively. Band intensities were calculated using Image J (NIH, USA). LC3 II band intensities were normalized according to β-Actin.

### Identification of differentially expressed miRNAs from public microarray data

To identify the differentially expressed miRNAs (DEMIs) significantly related to diabetic cardiomyopathy, the miRNA expression profile GSE44179 based on GPL14613 (miRNA-2_0) Affymetrix Multispecies miRNA-2_0 array was downloaded from the Gene Expression Omnibus (GEO) database (http://www.ncbi.nlm.nih.gov/geo/). The samples contained in GSE44179 were left ventricle (LV) myocardial tissues from a high-fat diet and streptozotocin (STZ)-induced diabetic (n = 4) and non-diabetic control (n = 2) Wistar rats^51^. Then, DEMIs between diabetic and control samples were screened out via the GEOquery and limma packages of R in which the expression data was fit to a linear model after filtering lowly-expressed genes with median expression level as a threshold and assigning relative quality weights for each sample^52^. The cut of criteria was set as adjusted p-value < 0.05 and |logFC| > 1. To further visualize the results of differential expression analysis, the heat map of hierarchical clustering based on Euclidean distance with complete linkage metric and volcano plot were constructed using the R packages pheatmap^53^; ggplot2^54^ and ggrepel^55^, respectively.

The potential target genes of DEMIs were predicted through TargetScan v7.2 (http://www.targetscan.org/vert_72/)^56^ and miRDB (http://mirdb.org)^57^ integrated into the miRWalk v2.0 (http://mirwalk.umm.uni-heidelberg.de/) predicted target module^58^ and then screened by Venn diagram to find out the common targets with those of rat miR-499-5p, miR-133a-5p, miR-133b-5p, which were identified by DIANA-micro T-CDS v5.0 with the miRNA target gene (miTG) prediction score threshold of 0.7 (http://www.microrna.gr/microT-CDS/)^59^. A regulatory network of miR-499-5p, miR-133a-5p, miR-133b-5p associated to those common targets were constructed using Cytoscape v3.8.2 (https://cytoscape.org). Gene Ontology (GO) and KEGG pathway enrichment of the predicted interactions were performed using the functional analysis mode of the Cytoscape plugin ClueGO v2.5.7^60^. The statistical significance of each term and pathway analyzed was calculated with a two-sided hypergeometric test and Bonferroni step-down *p*-value correction and a predefined kappa score level (≥ 0.4) was set to link them for a functionally grouped network (only annotations with the significance selection criteria as *p* ≤ 0.05 were shown). Raw data of GO-enriched functional annotations for the predicted targets of miR-499, miR-133a, and miR-133b given as “Supplementary file”. To understand through which ways the predicted targets of qRT-PCR-validated DEMIs are involved in diabetic cardiomyopathy pathogenesis and to validate those three miRNAs are truly the regulatory miRNAs of the diabetic cardiomyopathy-related genes, we generated a summary list by performing functional enrichment analysis with DAVID (DAVID; v6.8; https://david.ncifcrf.gov). GO terms with FDR-adjusted p-values < 0.1 were included among which we listed the GO terms according to their enrichment p-values (EASE score) and gene counts besides their relevance to the myocardial structural and functional changes in response to hyperglycemia.

### Statistical analysis

All data are expressed as ± standard deviation (± SD). Shapiro–Wilk test (α = 0.05)^61^ was performed to check the normal distribution using qqnorm in conjunction with the qqline functions and the Shapiro test of R version 4.0.3. Due to non-normal distribution in the values of tube formation and wound healing assays and qRT-PCR analysis non-parametric Mann–Whitney U tests were performed. ROS values that followed a normal distribution were compared using a t-test for independent groups. A value of p < 0.05 was considered statistically significant. Analysis was performed using GraphPad PRISM software (GraphPad Software Inc., San Diego, California).

## Supplementary Information

Supplementary Information 1.

Supplementary Information 2.

Supplementary Information 3.

Supplementary Figure 1.

Supplementary Table 1.

Supplementary Table 2.

## Data Availability

Data is available from the authors by request.
