# Supplementary material for: Cardioprotective effect of extracellular vesicles derived from ticagrelor-pretreated cardiomyocyte on hyperglycemic cardiomyocytes through alleviation of oxidative and endoplasmic reticulum stress
Source: Sci Rep. 2022 Apr 5;12:5651. doi: 10.1038/s41598-022-09627-6 (PMC8983723; doi:10.1038/s41598-022-09627-6)
Supplement: Supplementary file 1 — Supplementary Information 1. [file 41598_2022_9627_MOESM1_ESM.pdf]

Specimen Name: H9C2

H9C2-Control Exosome Unstained-1

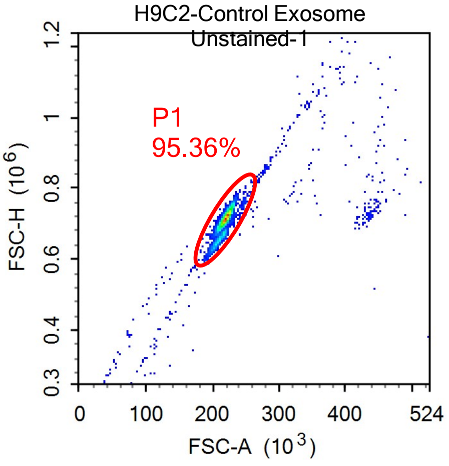

H9C2-Control Exosome Unstained-1 / P1

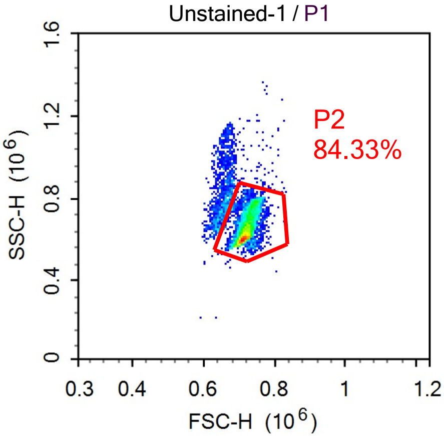

H9C2-Control Exosome Unstained-1 / P1 / P2

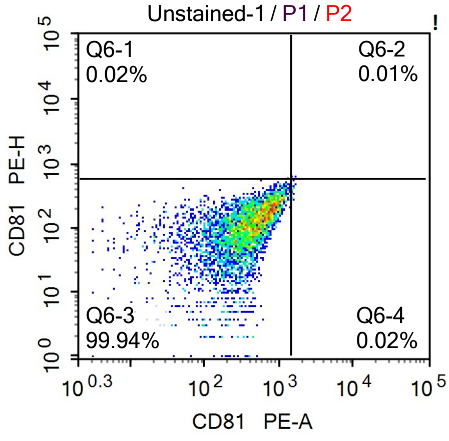

Gate	Count	% All	Mean X	Mean Y
All	10,000	100.00%	291,822	712,377
P1	9,536	95.36%	217,149	708,820

Gate	Count	% P1	Mean X	Mean Y
P1	9,536	100.00%	708,820	684,880
P2	8,042	84.33%	717,993	657,836

Gate	Count	% P2	Mean X	Mean Y
P2	8,042	100.00%	193	60
Q6-1	2	0.02%	1,383	608
Q6-2	1	0.01%	1,643	614
Q6-3	8,037	99.94%	192	60
Q6-4	2	0.02%	1,585	457

H9C2-Control Exosome CD81-1

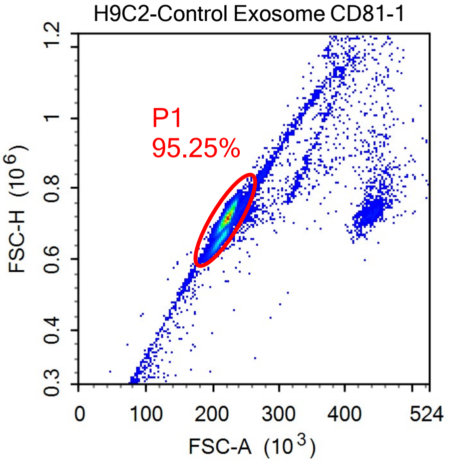

H9C2-Control Exosome CD81-1 / P1

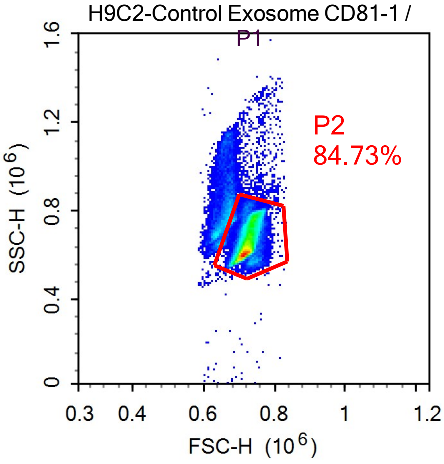

H9C2-Control Exosome CD81-1 / P1 / P2

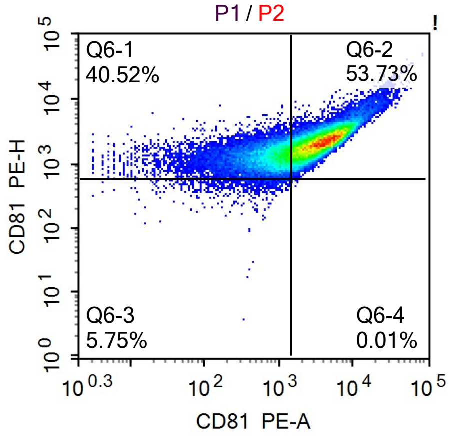

Gate	Count	% All	Mean X	Mean Y
All	100,000	100.00%	229,696	710,962
P1	95,250	95.25%	219,818	711,402

Gate	Count	% P1	Mean X	Mean Y
P1	95,250	100.00%	711,402	686,643
P2	80,707	84.73%	720,662	659,612

Gate	Count	% P2	Mean X	Mean Y
P2	80,707	100.00%	2,410	1,971
Q6-1	32,701	40.52%	78	1,366
Q6-2	43,361	53.73%	4,581	2,614
Q6-3	4,638	5.75%	-1,441	227
Q6-4	7	0.01%	1,656	531

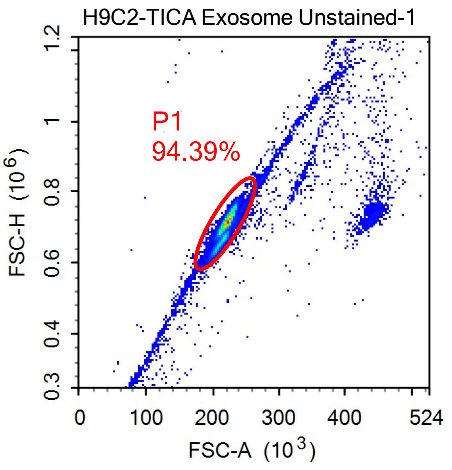

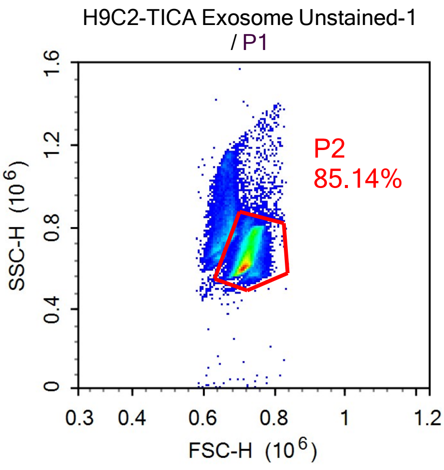

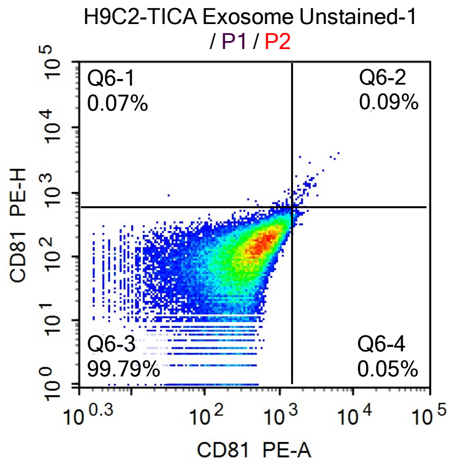

Gate	Count	% All	Mean X	Mean Y
All	100,000	100.00%	251,866	718,693
P1	94,385	94.39%	219,735	710,422

Gate	Count	% P1	Mean X	Mean Y
P1	94,385	100.00%	710,422	686,166
P2	80,359	85.14%	719,215	660,492

Gate	Count	% P2	Mean X	Mean Y
P2	80,359	100.00%	188	61
Q6-1	53	0.07%	1,032	689
Q6-2	71	0.09%	2,555	1,577
Q6-3	80,191	99.79%	185	59
Q6-4	44	0.05%	1,689	437

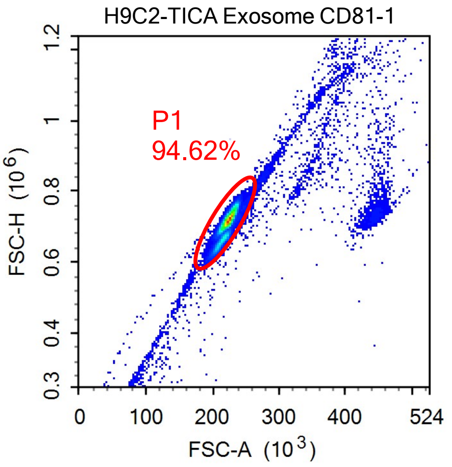

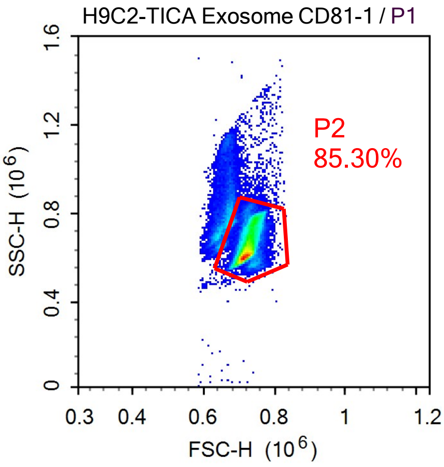

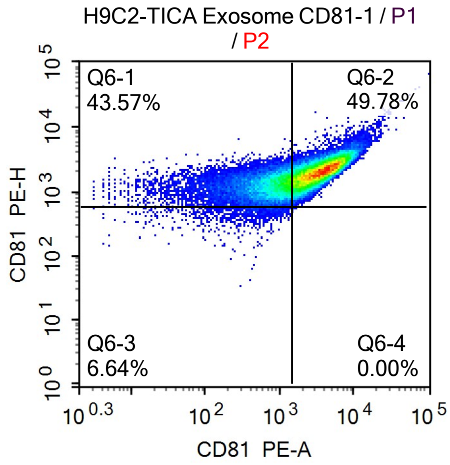

Gate	Count	% All	Mean X	Mean Y
All	100,000	100.00%	228,350	711,363
P1	94,617	94.62%	221,531	713,690

Gate	Count	% P1	Mean X	Mean Y
P1	94,617	100.00%	713,690	688,008
P2	80,710	85.30%	722,670	662,349

Gate	Count	% P2	Mean X	Mean Y
P2	80,710	100.00%	1,951	1,742
Q6-1	35,169	43.57%	102	1,314
Q6-2	40,181	49.78%	4,000	2,319
Q6-3	5,358	6.64%	-1,285	232
Q6-4	2	0.00%	1,579	556

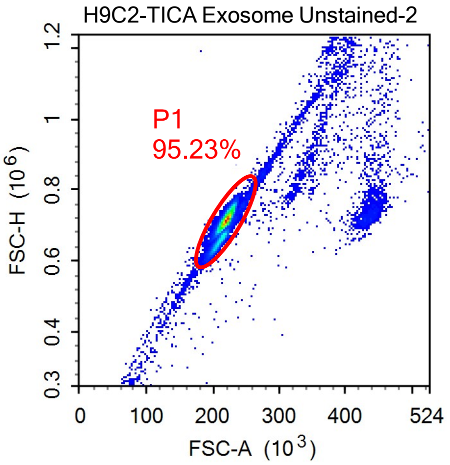

Gate	Count	% All	Mean X	Mean Y
All	100,000	100.00%	227,257	715,789
P1	95,228	95.23%	217,570	708,896

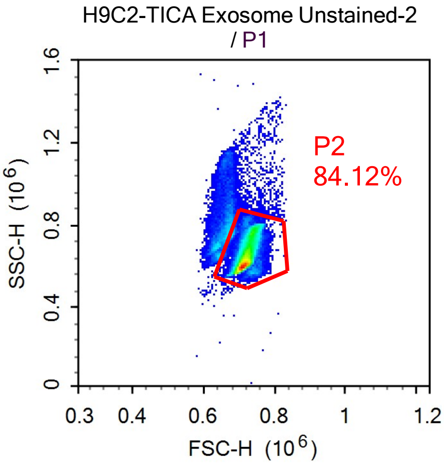

Gate	Count	% P1	Mean X	Mean Y
P1	95,228	100.00%	708,896	686,807
P2	80,102	84.12%	718,170	658,745

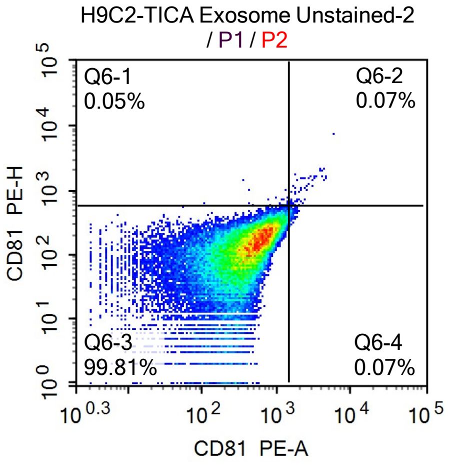

Gate	Count	% P2	Mean X	Mean Y
P2	80,102	100.00%	187	67
Q6-1	43	0.05%	1,096	673
Q6-2	53	0.07%	2,514	1,342
Q6-3	79,950	99.81%	184	66
Q6-4	56	0.07%	1,656	449

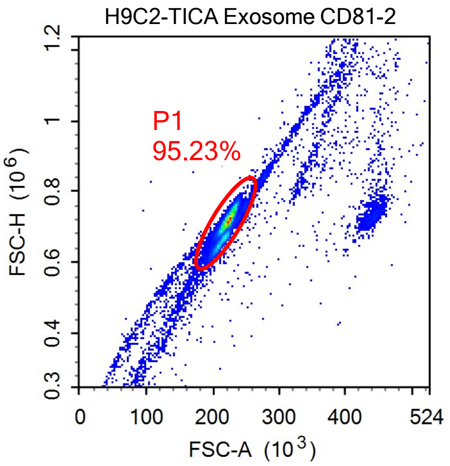

Gate	Count	% All	Mean X	Mean Y
All	100,000	100.00%	284,203	727,703
P1	95,231	95.23%	220,849	712,723

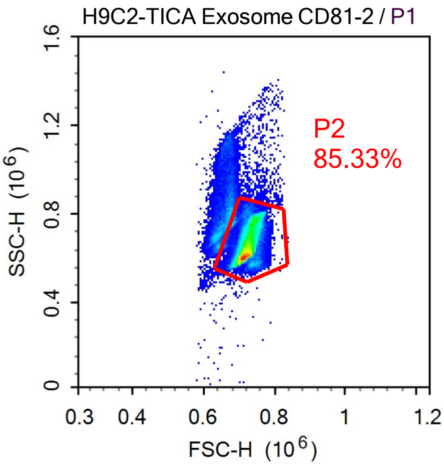

Gate	Count	% P1	Mean X	Mean Y
P1	95,231	100.00%	712,723	688,667
P2	81,259	85.33%	721,501	663,483

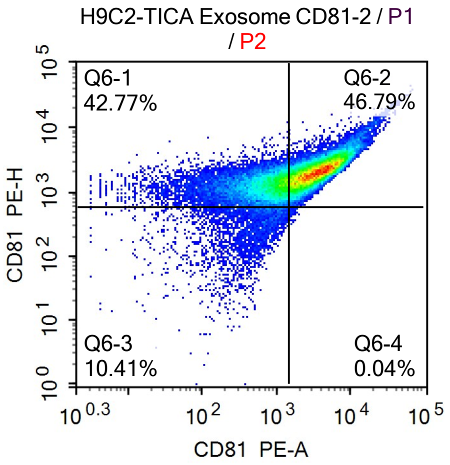

Gate	Count	% P2	Mean X	Mean Y
P2	81,259	100.00%	1,957	1,765
Q6-1	34,753	42.77%	51	1,331
Q6-2	38,020	46.79%	4,293	2,506
Q6-3	8,456	10.41%	-711	219
Q6-4	30	0.04%	1,677	483

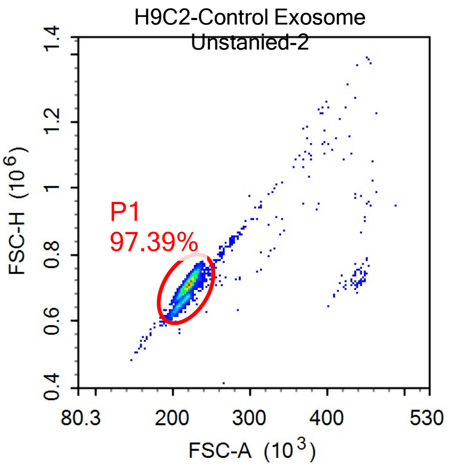

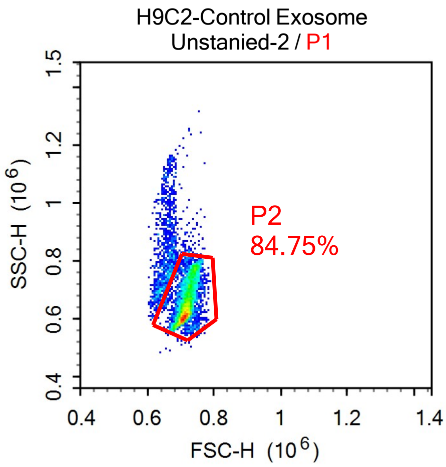

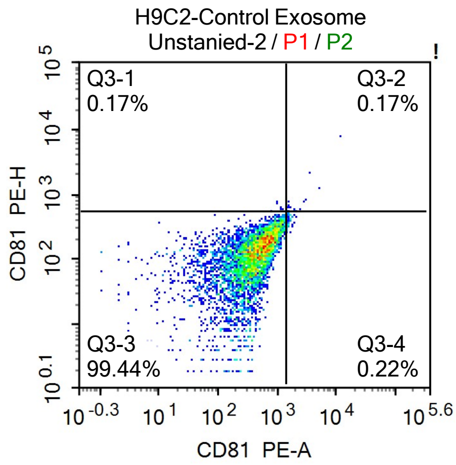

Gate	Count	% All	Mean X	Mean Y
All	10,000	100.00%	222,024	709,673
P1	9,739	97.39%	218,997	706,147

Gate	Count	% P1	Mean X	Mean Y
P1	9,739	100.00%	706,147	683,034
P2	8,254	84.75%	714,632	656,637

Gate	Count	% P2	Mean X	Mean Y
P2	8,254	100.00%	226	79
Q3-1	14	0.17%	1,238	634
Q3-2	14	0.17%	3,000	1,341
Q3-3	8,208	99.44%	217	76
Q3-4	18	0.22%	1,657	436

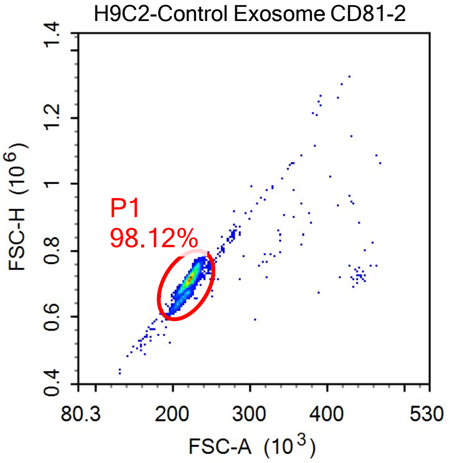

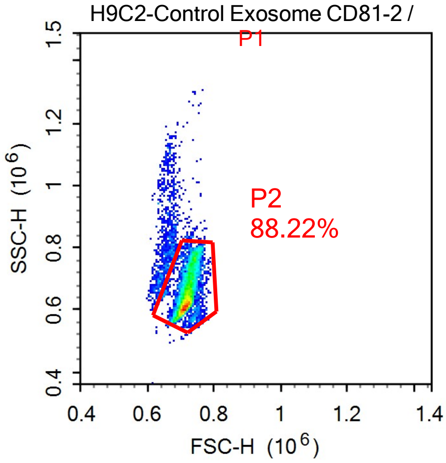

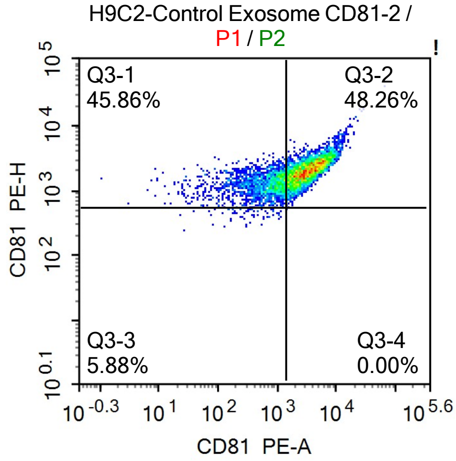

Gate	Count	% All	Mean X	Mean Y
All	10,000	100.00%	227,999	715,789
P1	9,812	98.12%	220,540	710,890

Gate	Count	% P1	Mean X	Mean Y
P1	9,812	100.00%	710,890	677,300
P2	8,656	88.22%	717,563	656,990

Gate	Count	% P2	Mean X	Mean Y
P2	8,656	100.00%	1,778	1,776
Q3-1	3,970	45.86%	-79	1,341
Q3-2	4,177	48.26%	3,967	2,383
Q3-3	509	5.88%	-1,700	195
Q3-4	0	0.00%	0	0

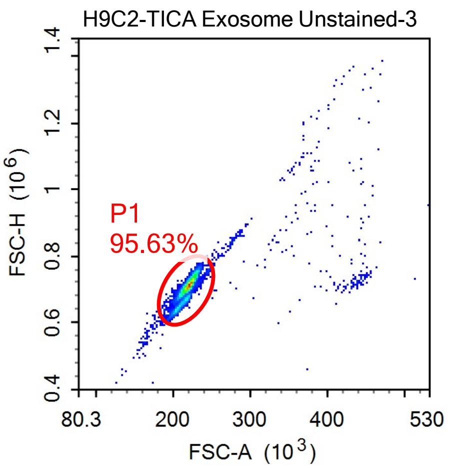

Gate	Count	% All	Mean X	Mean Y
All	10,000	100.00%	406,685	749,928
P1	9,563	95.63%	218,482	708,370

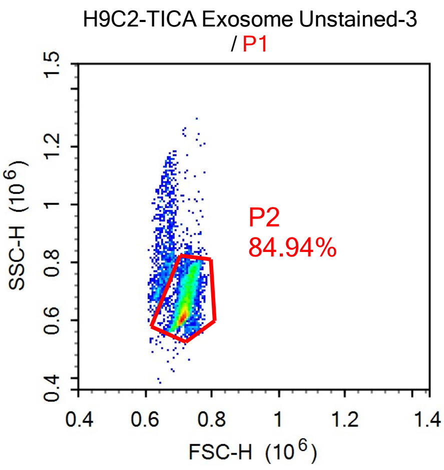

Gate	Count	% P1	Mean X	Mean Y
P1	9,563	100.00%	708,370	683,778
P2	8,123	84.94%	716,536	658,049

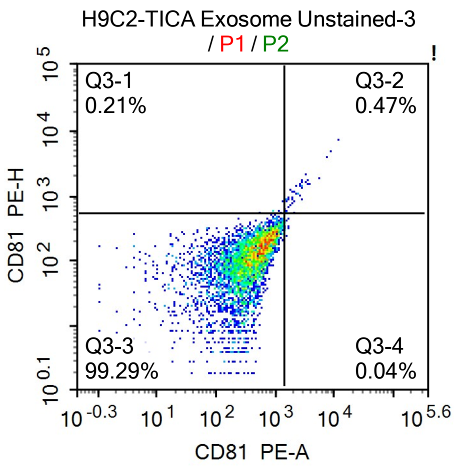

Gate	Count	% P2	Mean X	Mean Y
P2	8,123	100.00%	208	76
Q3-1	17	0.21%	1,180	659
Q3-2	38	0.47%	3,099	1,702
Q3-3	8,065	99.29%	192	67
Q3-4	3	0.04%	1,600	382

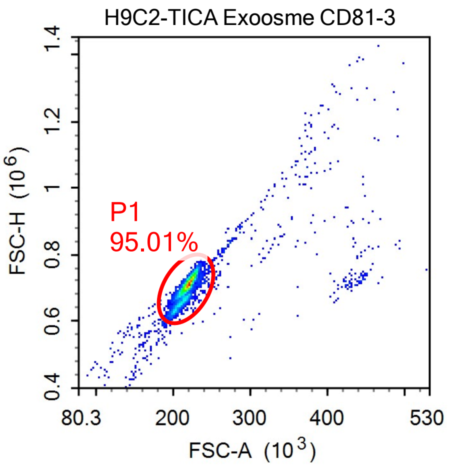

Gate	Count	% All	Mean X	Mean Y
All	10,000	100.00%	439,466	732,053
P1	9,501	95.01%	217,824	705,284

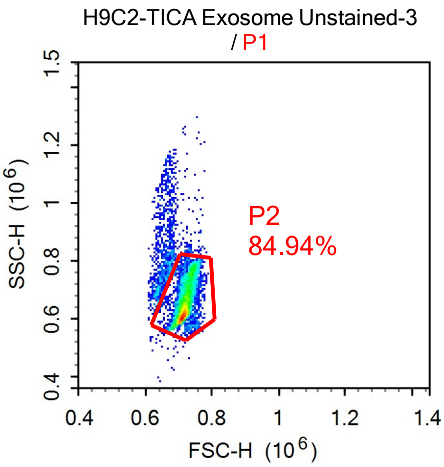

Gate	Count	% P1	Mean X	Mean Y
P1	9,563	100.00%	708,370	683,778
P2	8,123	84.94%	716,536	658,049

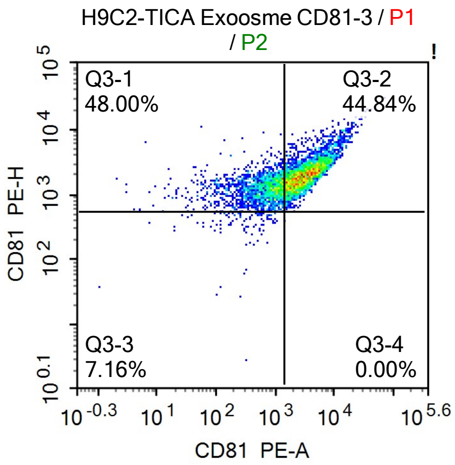

Gate	Count	% P2	Mean X	Mean Y
P2	7,989	100.00%	1,714	1,871
Q3-1	3,835	48.00%	-59	1,426
Q3-2	3,582	44.84%	4,150	2,620
Q3-3	572	7.16%	-1,658	157
Q3-4	0	0.00%	0	0

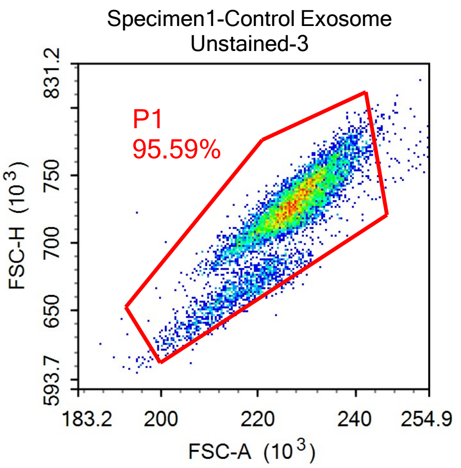

Gate	Count	% All	Mean X	Mean Y
All	10,000	100.00%	285,623	732,892
P1	9,559	95.59%	226,000	721,937

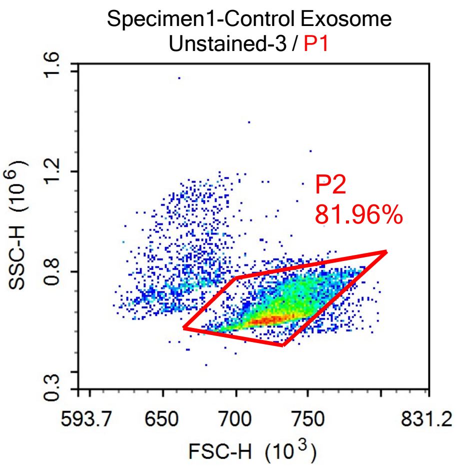

Gate	Count	% P1	Mean X	Mean Y
P1	9,559	100.00%	721,937	683,899
P2	7,835	81.96%	730,712	657,257

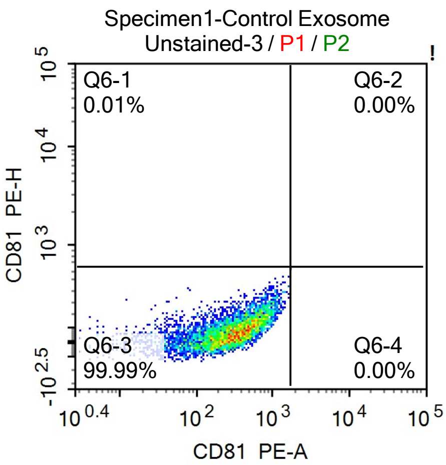

Gate	Count	% P2	Mean X	Mean Y
P2	7,835	100.00%	187	56
Q6-1	1	0.01%	1,687	646
Q6-2	0	0.00%	0	0
Q6-3	7,834	99.99%	186	56
Q6-4	0	0.00%	0	0

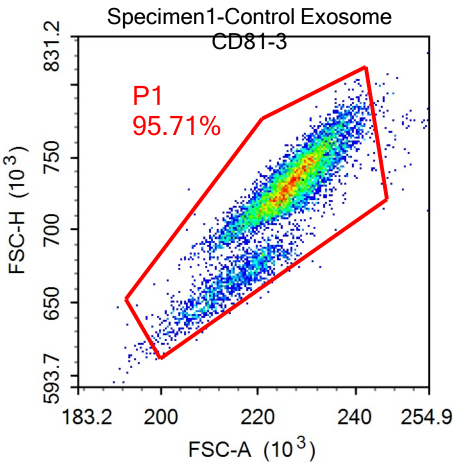

Gate	Count	% All	Mean X	Mean Y
All	10,000	100.00%	335,369	739,740
P1	9,571	95.71%	224,986	723,179

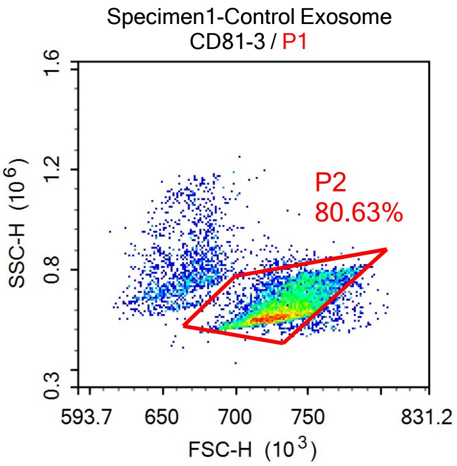

Gate	Count	% P1	Mean X	Mean Y
P1	9,571	100.00%	723,179	685,352
P2	7,717	80.63%	732,632	658,381

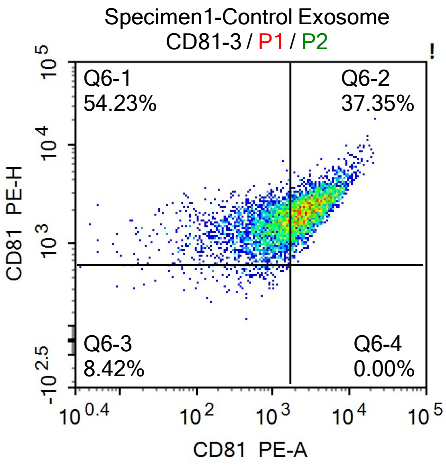

Gate	Count	% P2	Mean X	Mean Y
P2	7,717	100.00%	1,399	1,671
Q6-1	4,185	54.23%	129	1,409
Q6-2	2,882	37.35%	3,833	2,354
Q6-3	650	8.42%	-1,212	324
Q6-4	0	0.00%	0	0

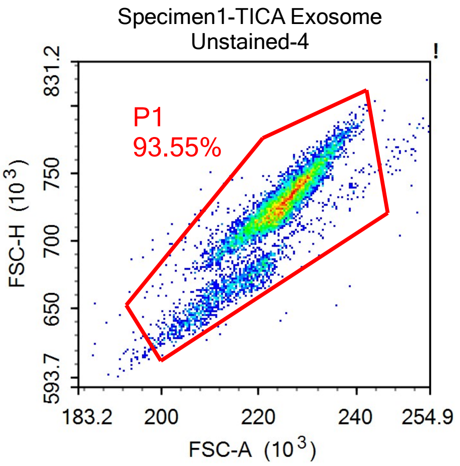

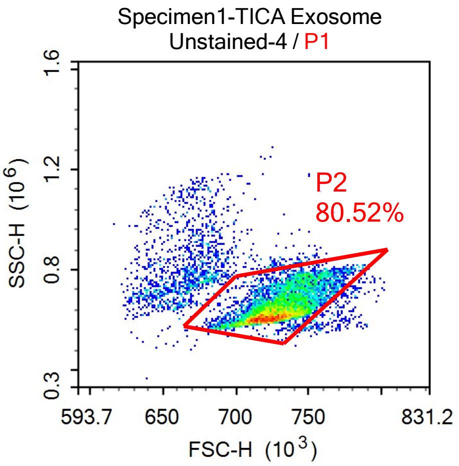

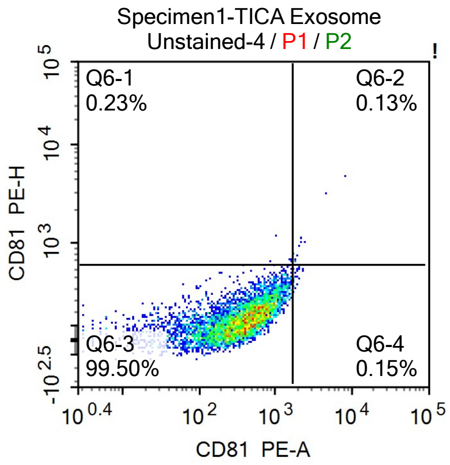

Gate	Count	% All	Mean X	Mean Y
All	10,000	100.00%	386,980	821,109
P1	9,355	93.55%	223,178	720,477

Gate	Count	% P1	Mean X	Mean Y
P1	9,355	100.00%	720,477	685,527
P2	7,533	80.52%	729,455	657,198

Gate	Count	% P2	Mean X	Mean Y
P2	7,533	100.00%	233	125
Q6-1	17	0.23%	958	774
Q6-2	10	0.13%	2,877	1,451
Q6-3	7,495	99.50%	226	122
Q6-4	11	0.15%	1,958	547

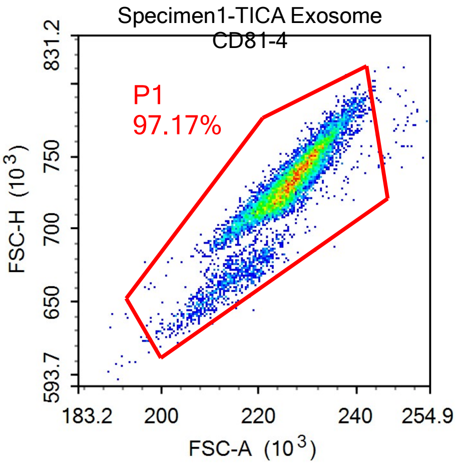

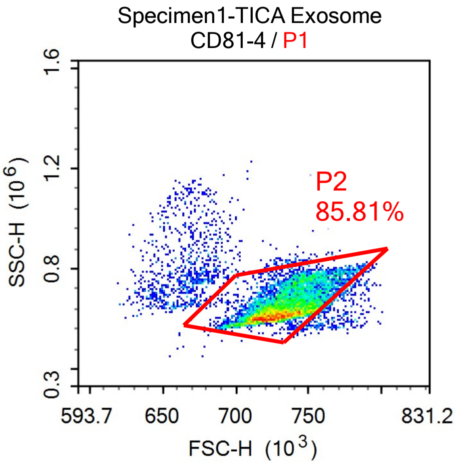

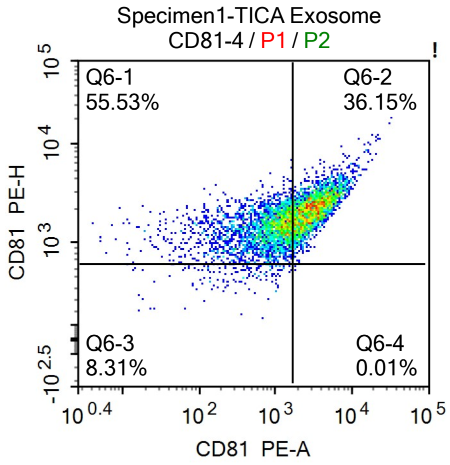

Gate	Count	% All	Mean X	Mean Y
All	10,000	100.00%	263,058	734,808
P1	9,717	97.17%	225,541	728,150

Gate	Count	% P1	Mean X	Mean Y
P1	9,717	100.00%	728,150	675,961
P2	8,338	85.81%	733,570	658,877

Gate	Count	% P2	Mean X	Mean Y
P2	8,338	100.00%	1,386	1,612
Q6-1	4,630	55.53%	201	1,355
Q6-2	3,014	36.15%	3,807	2,306
Q6-3	693	8.31%	-1,219	317
Q6-4	1	0.01%	1,839	597

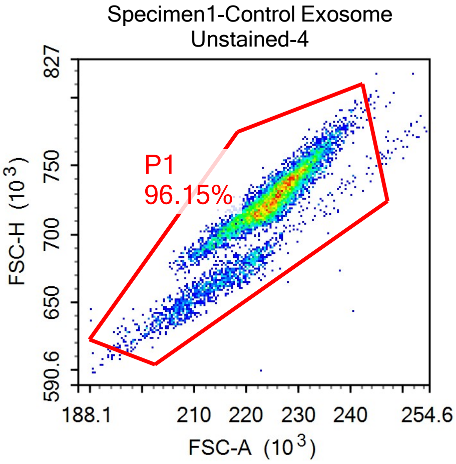

Gate	Count	% All	Mean X	Mean Y
All	10,000	100.00%	263,625	731,257
P1	9,615	96.15%	223,250	718,195

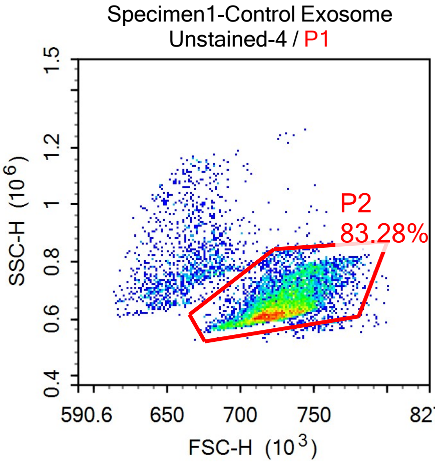

Gate	Count	% P1	Mean X	Mean Y
P1	9,615	100.00%	718,195	683,971
P2	8,007	83.28%	727,521	658,791

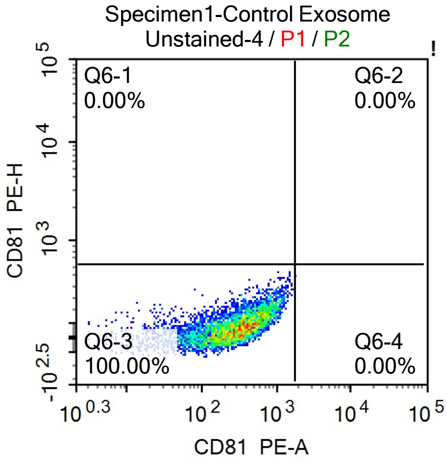

Gate	Count	% P2	Mean X	Mean Y
P2	8,007	100.00%	179	54
Q6-1	0	0.00%	0	0
Q6-2	0	0.00%	0	0
Q6-3	8,007	100.00%	179	54
Q6-4	0	0.00%	0	0

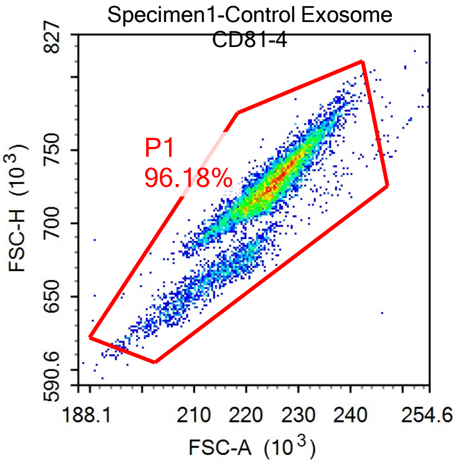

Gate	Count	% All	Mean X	Mean Y
All	10,000	100.00%	319,910	736,959
P1	9,618	96.18%	223,198	718,288

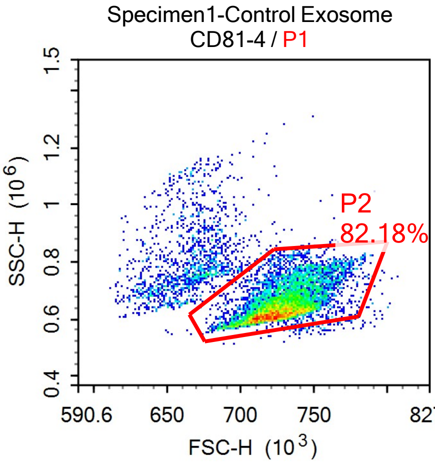

Gate	Count	% P1	Mean X	Mean Y
P1	9,618	100.00%	718,288	684,568
P2	7,904	82.18%	728,231	658,361

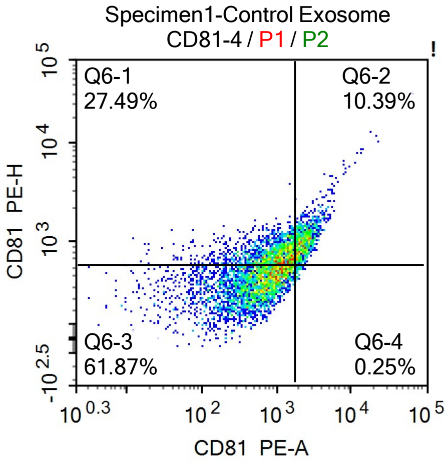

Gate	Count	% P2	Mean X	Mean Y
P2	7,904	100.00%	624	594
Q6-1	2,173	27.49%	762	823
Q6-2	821	10.39%	3,196	1,551
Q6-3	4,890	61.87%	125	332
Q6-4	20	0.25%	2,000	557

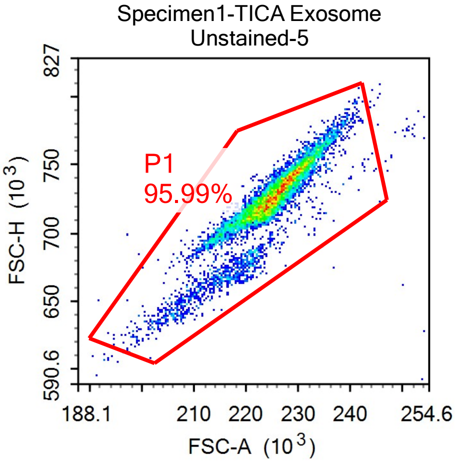

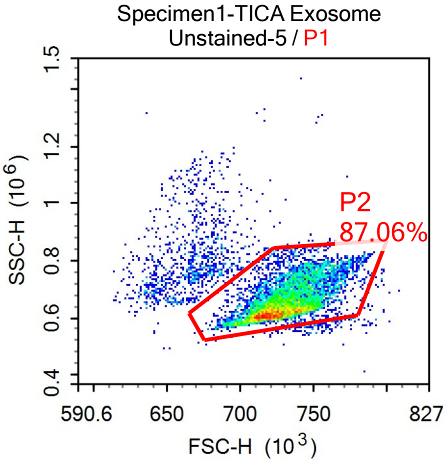

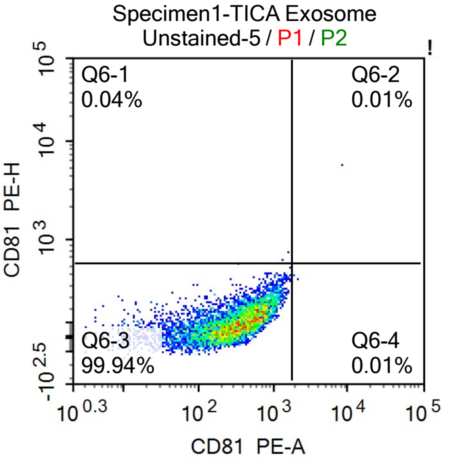

Gate	Count	% All	Mean X	Mean Y
All	10,000	100.00%	367,504	751,607
P1	9,599	95.99%	224,170	722,907

Gate	Count	% P1	Mean X	Mean Y
P1	9,599	100.00%	722,907	677,257
P2	8,357	87.06%	729,598	658,824

Gate	Count	% P2	Mean X	Mean Y
P2	8,357	100.00%	199	78
Q6-1	3	0.04%	1,066	701
Q6-2	1	0.01%	8,112	5,612
Q6-3	8,352	99.94%	198	77
Q6-4	1	0.01%	2,036	455

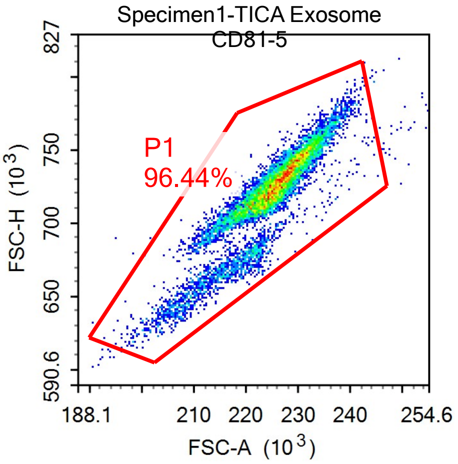

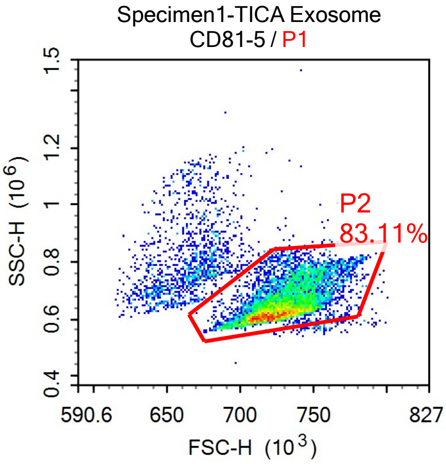

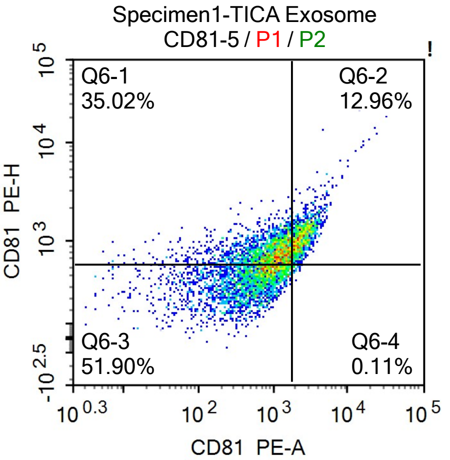

Gate	Count	% All	Mean X	Mean Y
All	10,000	100.00%	264,776	739,076
P1	9,644	96.44%	224,131	719,079

Gate	Count	% P1	Mean X	Mean Y
P1	9,644	100.00%	719,079	685,025
P2	8,015	83.11%	728,639	659,762

Gate	Count	% P2	Mean X	Mean Y
P2	8,015	100.00%	629	663
Q6-1	2,807	35.02%	684	854
Q6-2	1,039	12.96%	2,962	1,431
Q6-3	4,160	51.90%	6	343
Q6-4	9	0.11%	2,008	568
