# Supplementary figures and images for: Cardioprotective effect of extracellular vesicles derived from ticagrelor-pretreated cardiomyocyte on hyperglycemic cardiomyocytes through alleviation of oxidative and endoplasmic reticulum stress
Source: Sci Rep. 2022 Apr 5;12:5651. doi: 10.1038/s41598-022-09627-6 (PMC8983723; doi:10.1038/s41598-022-09627-6)

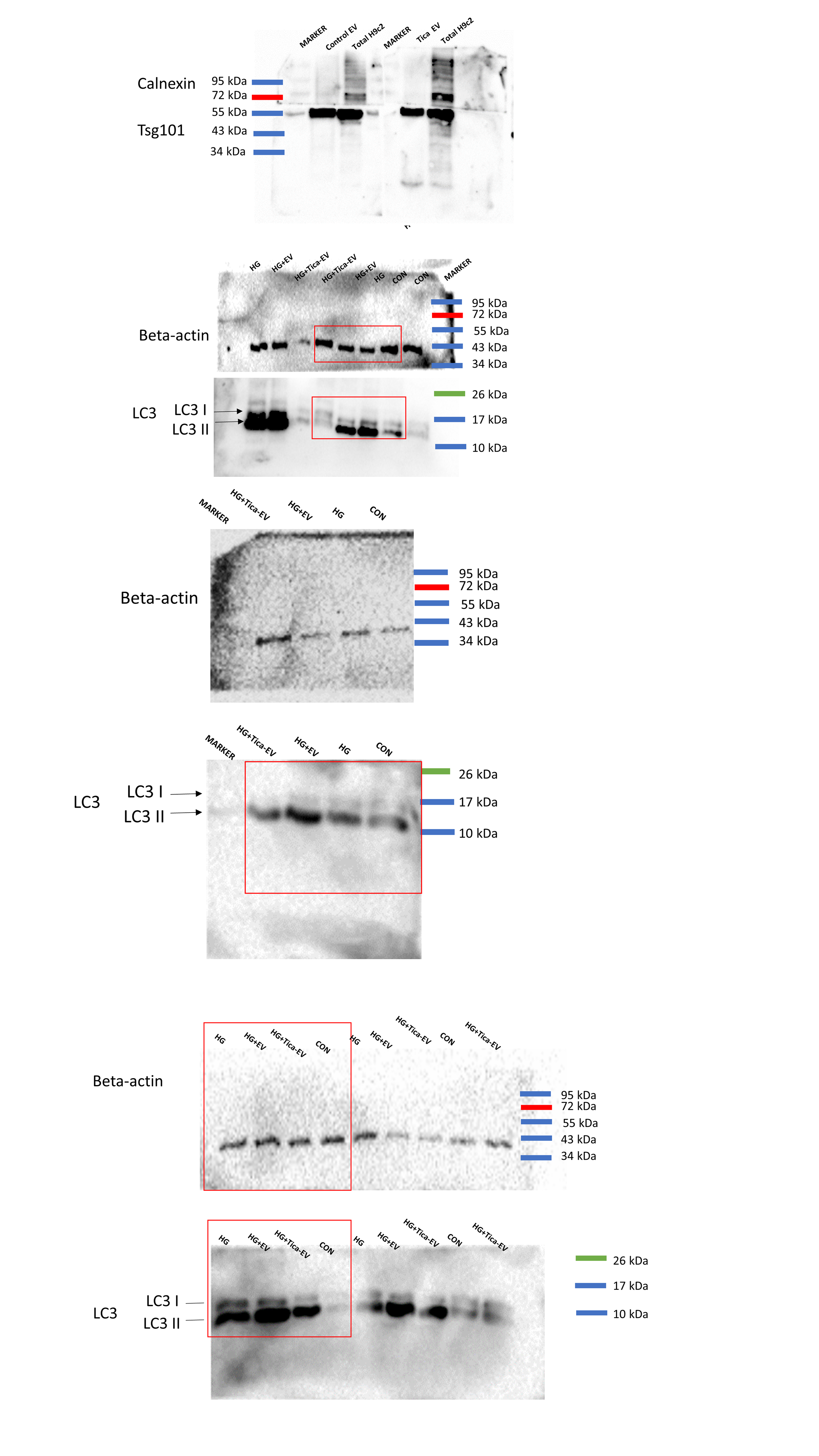

Supplement: Supplementary file 3 — Supplementary Information 3. [file 41598_2022_9627_MOESM3_ESM.tif]

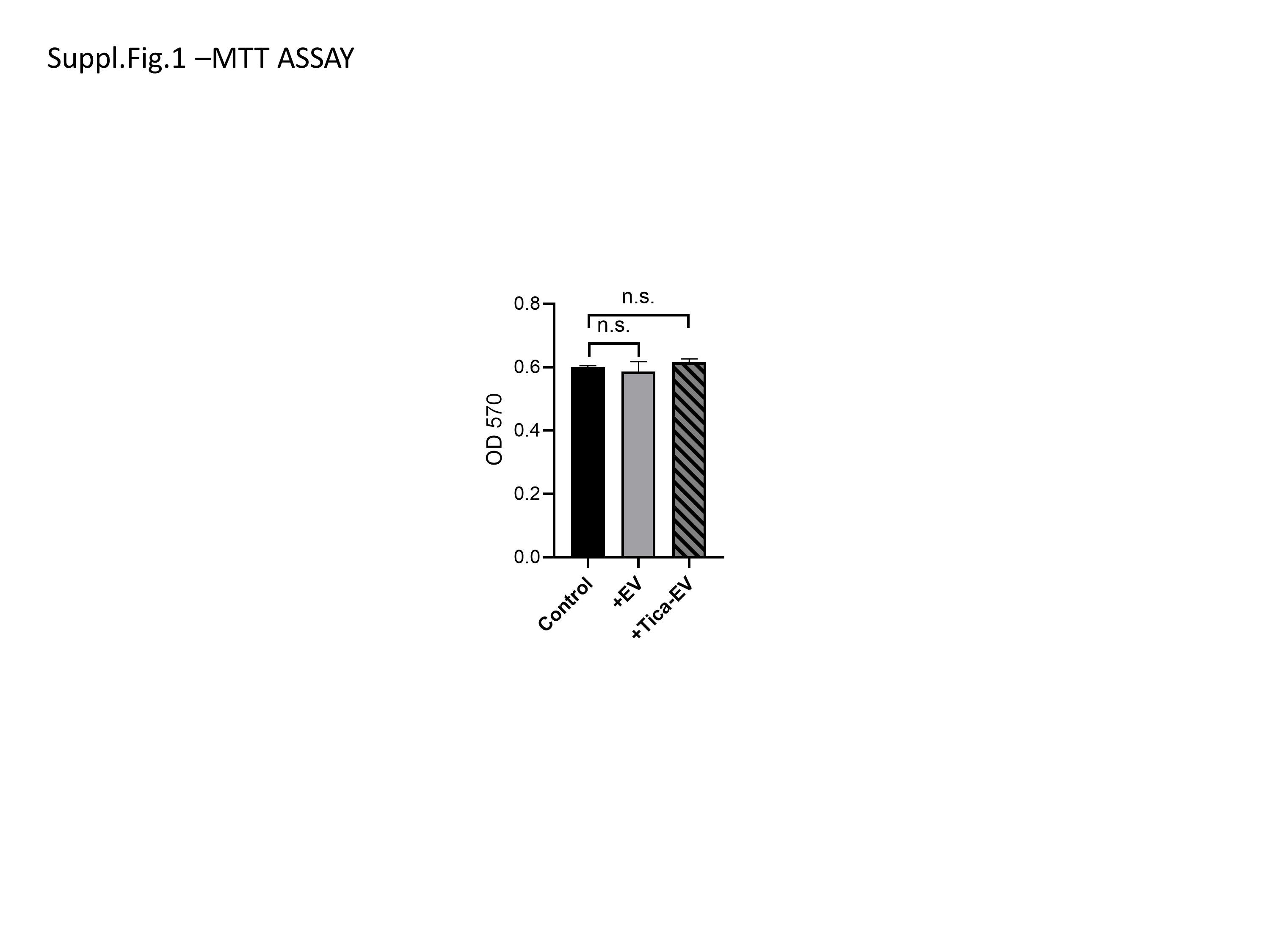

Supplement: Supplementary file 4 — Supplementary Figure 1. [file 41598_2022_9627_MOESM4_ESM.tif]
