# Supplementary material for: Cardioprotective effect of extracellular vesicles derived from ticagrelor-pretreated cardiomyocyte on hyperglycemic cardiomyocytes through alleviation of oxidative and endoplasmic reticulum stress
Source: Sci Rep. 2022 Apr 5;12:5651. doi: 10.1038/s41598-022-09627-6 (PMC8983723; doi:10.1038/s41598-022-09627-6)
Supplement: Supplementary file 5 — Supplementary Table 1. [file 41598_2022_9627_MOESM5_ESM.docx]

**Supplementary Table 1: The primers designed for qRT-PCR.**

| **Names of miRNA/Gene** | **Forward Primers** | **Reverse Primers** |
| --- | --- | --- |
| miR-499-5p | 5’ - GAT TAA TTA AGA CTT GCA GTG CA -3’ | 5′ - CCA GTG CAG GGT CCG AGG TA -3′ |
| miR-133a-5p | 5’ - GAT GAG AGC TGG TAA AAT GG -3’ | 5′- CCA GTG CAG GGT CCG AGG TA -3′ |
| miR-133b-5p | 5’ - TAA TAA GCT GGT CAA ACG GA -3’ | 5′- CCA GTG CAG GGT CCG AGG TA -3′ |
| U6 | 5’ – GCT TCG GCA GCA CAT ATA CTA AAA T -3’ | 5’ - CGC TTC ACG AAT TTG CGT GTC AT -3’ |
| Bcl2 | 5’ – CCT GGC ATC TTC TCC TTC -3’ | 5’ -TGCT GAC CTC ACT TGT GG -3’ |
| Bax | 5’ – AAT CAT GGA CGG GTC C -3’ | 5’ - GCC CAT CTT CTT CCA G -3’ |
| Grp78 | 5’ - CAT AGC CAA CGA TCA GGG CA GAA -3’ | 5’ - AAG GGT CAT TCC AAG TGC GT -3’ |
| Calregulin | 5’ - CGC AGA CCC TGC CAT CTA T -3’ | 5’ - GGT CCC CGT AGA ATT TGC CA -3’ |
| ENT1 | 5’ - TGG TTA CTT CAT CAC GGC CT -3’ | 5’ - TTG GCT CCT CTC CTT CAC TG -3’ |
| Beclin | 5’ - TGT TTG GAG ATG TTG GAG CA -3’ | 5’ - TCG CTG GTA CTG AGC TTC CT -3’ |
| Bnip3 | 5’ - GAA TCT GGA CGA AGC AGC TC -3’ | 5’ - AAC ATT TTC TGG CCG ACT TG -3’ |
| Cyclophilin | 5’ - GGG AAG GTG AAA GAA GGC AT -3’ | 5’ - GAG AGC AGA GAT TAC AGG G -3’ |
