# Supplementary material for: Cardioprotective effect of extracellular vesicles derived from ticagrelor-pretreated cardiomyocyte on hyperglycemic cardiomyocytes through alleviation of oxidative and endoplasmic reticulum stress
Source: Sci Rep. 2022 Apr 5;12:5651. doi: 10.1038/s41598-022-09627-6 (PMC8983723; doi:10.1038/s41598-022-09627-6)
Supplement: Supplementary file 6 — Supplementary Table 2. [file 41598_2022_9627_MOESM6_ESM.docx]

Supplementary Table 2: The stem-loop primers designed for miRNA-qRT-PCR.

| **Names of miRNAs** | **Stem-loop primers** |
| --- | --- |
| miR-499-5p | 5′- GTC GTA TCC AGT GCA GGG TCC GAG GTA TTC GCA CTG GAT ACG AC AAA CAT CAC TG- 3’ |
| miR-133a-5p | 5′- GTC GTA TCC AGT GCA GGG TCC GAG GTA TTC GCA CTG GAT ACG AC ATT TGG TTC- 3’ |
| miR.-133b-5p | 5′- GTC GTA TCC AGT GCA GGG TCC GAG GTA TTC GCA CTG GAT ACG AC ACT TGG TTC- 3’ |
